# Microwave-Driven Synthesis of Iron-Oxide Nanoparticles for Molecular Imaging

**DOI:** 10.3390/molecules24071224

**Published:** 2019-03-28

**Authors:** Irene Fernández-Barahona, Maria Muñoz-Hernando, Fernando Herranz

**Affiliations:** 1 NanoMedMol Group, Instituto de Química Médica, Consejo Superior de Investigaciones Científicas (CSIC) and CIBERES, C/Juan de la Cierva 3, 28006 Madrid, Spain; ifernandezbarahona@ucm.es (I.F.-B.); maria.munoz@cnic.es (M.M.-H.); 2 Facultad de Farmacia, Universidad Complutense de Madrid, Plaza de ramón y Cajal, 28040 Madrid, Spain; 3 Centro Nacional de Investigaciones Cardiovasculares Carlos III (CNIC), C/Melchor Fernández-Almagro 3, 28029 Madrid, Spain

**Keywords:** iron-oxide nanoparticles, molecular imaging, magnetic resonance imaging (MRI), positron-emission tomography (PET)

## Abstract

Here, we present a comprehensive review on the use of microwave chemistry for the synthesis of iron-oxide nanoparticles focused on molecular imaging. We provide a brief introduction on molecular imaging, the applications of iron oxide in biomedicine, and traditional methods for the synthesis of these nanoparticles. The review then focuses on the different examples published where the use of microwaves is key for the production of nanoparticles. We study how the different parameters modulate nanoparticle properties, particularly for imaging applications. Finally, we explore principal applications in imaging of microwave-produced iron-oxide nanoparticles.

## 1. Introduction

The use of microwaves in chemistry implies the application of electromagnetic radiation, with wavelengths from 1 m to 1 cm, to heat a solution in a rapid and homogeneous manner. Most commercially used microwaves work at a frequency of 2.45 GHz. The usefulness of microwave (MW) chemistry in organic chemistry is recognized since early examples by Giguere in the 1980s [1]. In organic synthesis, the most important aspect of using microwaves is the dramatic enhancement of reaction rates. For example, the Suzuki–Miyaura cross-coupling goes from several hours of reaction time, with traditional heating, to less than a minute when using MW [2]. However, there are many more advantages of using MWs in chemistry such as a reduction in energy use, the development of fully automated processes, the development of new materials, its use in polymer syntheses, and its contribution to the chemical industry.

The use of MW for the production of nanoparticles (NPs) is more recent but shares all the mentioned advantages. One of these advantages is particularly important for the synthesis of NPs—heating homogeneity. Compared to traditional heating, the use of MW ensures a uniform heat distribution of the heat in the solution, while, in traditional methods, the heating tends to be heterogeneous and focused on the reaction flask walls. This homogeneous heating directly translates into a narrow size distribution and controlled physicochemical properties in the nanomaterial. The microwave synthesis of nanomaterials was dynamically developed in recent years, mainly thanks to the appearance of new professional microwave reactors [3]. 

In spite of a recent development, the field of MW synthesis of NPs is vast. Here, we focus on a particular aspect especially relevant for biomedical applications: the microwave synthesis of iron-oxide NPs for molecular imaging. Before further inquiring into this topic, we provide a brief introduction on molecular imaging, as well as the main techniques and some of their features. We also introduce the use of iron-oxide nanoparticles (IONPs) in molecular imaging, and we explain why this nanomaterial is now one of the most used in biomedical imaging.

### 1.1. Molecular Imaging

Molecular imaging (MI) is defined as the ability to visualize and quantitatively measure the biochemical processes in a living organism at a cellular and molecular level. One of the main motivations of MI is to translate common in vitro bioassay strategies into an in vivo setting in an attempt to overcome existing limitations. During in vitro assays, the evaluation of entire intact organisms over time cannot be carried out. MI allows the non-invasive study of cells in their natural environment, providing a wide range of techniques capable of tracing cell movement, thus making it possible to perceive dynamic biological processes. Furthermore, MI enables longitudinal monitoring of subjects, facilitating long-term observations that allow elucidating specific behaviors, efficacy, and failure causes of treatments. These properties make MI a field with enormous potential and a large variety of applications, including diagnostics, drug discovery and development, theranostics, and personalized medicine.

**Figure 1:**
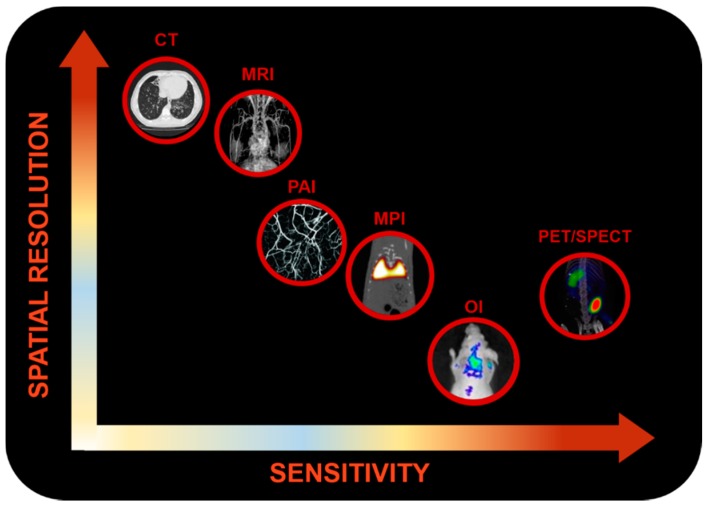
Graphical representation of different molecular imaging (MI) techniques classified by their sensitivity and spatial resolution. Computed tomography (CT) image adapted with permission from Reference [27]. Photoacoustic imaging (PAI) image adapted with permission from Reference [28]. Magnetic particle imaging (MPI) image adapted with permission from Reference [22]. Optical imaging (OI) image adapted with permission from Reference [29]. Positron-emission tomography (PET) image adapted with permission from Reference [30].

There is a broad array of MI modalities (Figure 1). Deciding which one should be used depends on the biochemical processes that are to be observed, together with the type of imaging data that needs to be acquired. Some currently used techniques are described below.

#### 1.1.1. Optical Imaging

The visualization of cells and tissues using light was used throughout the years in medical diagnostic imaging. Several microscopy techniques were developed for the evaluation of in vitro and ex vivo samples. Nevertheless, alongside these techniques, a number of macroscopic imaging modalities, which allow non-invasive, repetitive, and whole-body imaging of small animals, emerged. Optical imaging (OI) techniques are capable of achieving high imaging sensitivities and involve the detection of low-energy photons, making them relatively safe. However, the latter characteristic limits their depth of penetration to only a few centimeters, restricting their use to small-animal preclinical studies. Out of these, the most used are fluorescence and bioluminescence imaging. Fluorescence imaging uses fluorescent materials, such as proteins and dyes, as labels to visualize different molecular processes and structures. During the process, following the administration of the fluorescent imaging agent, an excitation light of appropriate wavelength is used to illuminate the subject. This leads to the excitation of the fluorophore and the subsequent emission of light. This light is detected and posteriorly converted into an image that provides its location within the subject.

One of the major challenges in this technique is to overcome the attenuation and scattering of light. For that purpose, fluorescent imaging agents that emit in the near-infrared (NIR) spectrum are used. This type of fluorophore can be imaged at higher depths and, furthermore, increases imaging sensitivity by decreasing tissue autofluorescence. Fluorescence imaging is used in different preclinical MI applications such as tumor detection [4], the study of specific biochemical processes [5], and the study of neurodegenerative diseases [6]. 

#### 1.1.2. Photoacoustic Imaging

Photoacoustic imaging (PAI) is an emerging modality based on the photoacoustic effect where light is converted into ultrasound waves that are detected outside the subject of interest. This imaging modality overcomes to a great extent the resolution and depth limitations of optical imaging while maintaining relatively high contrast. PAI is used to visualize tissues where intrinsic contrast is available, such as blood-vessel structure [7]. However, since many diseases do not manifest an endogenous photoacoustic contrast, it is essential to develop exogenous photoacoustic contrast agents that have a superior ability to absorb light and produce a stronger PA signal while being able to target diseased tissues. Imaging agents that are used for this purpose include single-walled carbon nanotubes (SWNT) for tumor targeting [8] and gold nanoparticles (AuNPs) for sentinel lymph-node targeting [9]. Nevertheless, PAI has some limitations, such as the inability of imaging bone or air structures, the lack of a multiplexing possibility, and the need for instrument–subject coupling.

#### 1.1.3. Nuclear Imaging

Positron-emission tomography (PET) and single-photon emission computed tomography (SPECT) are radionuclide MI techniques that enable the evaluation of biochemical changes within a living subject. These techniques possess unlimited depth of penetration with high sensitivity.

In PET, the system detects pairs of gamma rays (511 keV in energy and traveling in opposite directions to one another) emitted indirectly by a positron-emitting isotope. This radionuclide is introduced into the body by means of a biologically active molecule forming, which is known as tracer probe. This tracer is specific and selective to the target of interest. After this, computer analysis of tracer presence renders three-dimensional (3D) images. Radionuclides used in PET scanning are typically isotopes with short half-lives such as carbon-11 (^11^C), nitrogen-13 (^13^N), oxygen-15 (^15^O), fluorine-18 (^18^F), gallium-68 (^68^Ga), zirconium-89 (^89^Zr), or rubidium-82 (^82^Rb). Most of these radionuclides need to be produced using a cyclotron, constituting a disadvantage for the technique. For this reason, current research focusing on PET radionuclides that can be produced using a generator, such as ^68^Ga, is largely growing [10]. This change offers a cost-effective alternative for PET studies while requiring minimum space. In the clinic, PET is mainly used to image cancer through the use of [^18^F]-2-fluoro-2-deoxy-glucose ([^18^F]FDG). In addition, PET is commonly used in a wide range of applications in preclinical studies [11]. Some of them include tumor visualization [12,13], atherosclerosis detection [14,15], or the evaluation of the biodistribution of new pharmaceuticals [16], among others.

#### 1.1.4. Magnetic Resonance Imaging

Magnetic resonance imaging (MRI) is a technique that uses a powerful magnetic field and radio waves to visualize the internal structure and soft tissue morphology of the body. Contrast between different tissues in MR images can be generated via several ways but the different relaxation times of each tissue (longitudinal (T_1_) and transverse (T_2_) relaxation times) are the most commonly used and the most relevant in the context of MI. T_1_-weighted images are used to visualize anatomy. In these images, areas where the contrast agent is localized appear brighter than surrounding tissue due to the increased relaxation rate value (*r*_1_ = 1/T_1_). On the contrary, in T_2_-weighted images, due to a faster relaxation of transverse magnetization, areas where the contrast agent is accumulated appear darker than surrounding tissues in a T_2_-weighted sequence.

MRI is mainly used as an anatomical tool; however, the use of targeted imaging probes allows for its use in MI where relevant functional information can be gathered together with the unparalleled anatomical resolution of the technique.

Probe-targeted MRI, initially focused only on gadolinium (Gd) chelates, is now also possible with a wide variety of NPs. This is particularly true for iron-oxide nanoparticles, which are gaining much more attention, especially due to the toxicity concerns about Gd compounds. Some applications include detection of atherosclerosis [17,18] and visualization of tumors [19]. The main advantages of MRI are the lack of ionizing radiation, unlimited depth of penetration, and high spatial resolution. A major limitation is its poor sensitivity compared to other MI modalities, together with the long image acquisition times.

#### 1.1.5. Magnetic Particle Imaging

Magnetic particle imaging (MPI) is a novel technique that exploits the nonlinear magnetization of iron-based NPs to generate maps of tracer distribution [20]. This technique is capable of visualizing iron-based nanoparticles via direct induction; hence, its signal increases linearly with the amount of tracer present in the imaged volume [10]. For these reasons, MPI can be seen as a “hotspot” imaging technique with no background signal, similar to nuclear imaging techniques but without ionizing radiation. In addition, its magnetic signal does not suffer attenuation with depth. Research groups working with MPI and superparamagnetic IONPs already demonstrated their capability to track stem cells in small-animal models [21,22], and to visualize brain and tumor vasculature for potential diagnosis of stroke and cancer [23]. This technique is still under development; however, it has tremendous potential to surpass some of the limitations of IONPs in MRI without the inconvenience associated with nuclear imaging techniques [10]. 

Currently, research on multimodality imaging, a combination of different imaging modalities used at the same time, is gaining great interest. This approach offers a good solution to overcome some of the limitations of the independent techniques and provides larger amounts of information for a single experiment. Ideally, multimodality imaging would provide functional and molecular information (OI, PET, SPECT, and MPI) projected on datasets with anatomical information (computed tomography (CT), MRI). In addition, given the attention that this topic received, the development of multimodal contrast agents capable of giving signal in several MI modalities at the same time increased [24,25,26]. 

### 1.2. Iron-Oxide Nanoparticles for Molecular Imaging

IONPs are among the most versatile nanomaterials for biomedical applications. They are used for therapeutic purposes, such as hyperthermia, drug delivery, or antimicrobials, as well as in molecular imaging. In MI, their use covers practically all available techniques with examples in optical imaging, optoacoustic imaging [31], CT [32], positron emission tomography [30,33], and MRI [34,35]. Finally, they are key in the recently developed technique, MPI, where the superparamagnetic properties they often exhibit are used to trace the nanoparticles in this hotspot imaging technique [36]. The predominant use of IONPs for MI is based on three aspects that, although shared by several nanomaterials, are particularly prominent in IONPs: multifunctionalization, reproducibility, and tailored size.

**Figure 2:**
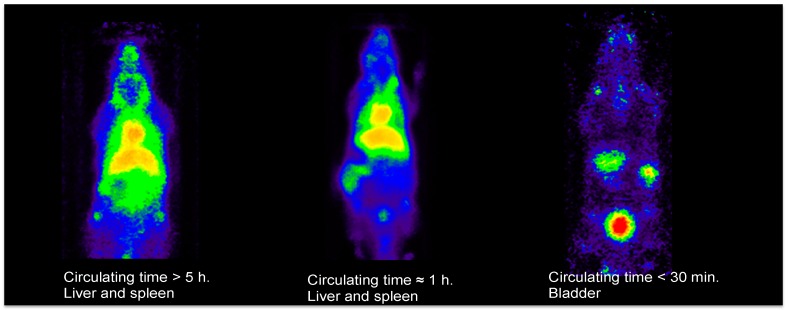
Differences in blood circulating time of ^68^Ga core-doped iron-oxide nanoparticles (IONPs) as a function of the hydrodynamic size (unpublished results by the authors).

Multifunctionalization is one of the most commonly cited properties of nanomaterials. However, if we understand it as the stable link between the surface of the nanoparticle and two or more molecules or biomolecules, the number of examples where this is achieved is not as large as one might expect. In the case of IONPs, the chemical variety of the coatings in use allows the attachment of many different chemical bonds ensuring access to a large molecular diversity. Reproducibility is key in science; however, when dealing with chemicals whose properties are size-dependent, it is of paramount importance to pay enough attention to this aspect. The different methods of producing IONPs certainly evolved to provide such necessary control. In particular, the use of MW-driven synthesis is especially efficient in this aspect as we show below. The final aspect, tailored size, is related to both the controlled synthesis and the reproducibility of the NPs. Thanks to this control, the pharmacokinetics of the IONP, the biodistribution, and the imaging properties can be finely tuned to each particular necessity to an extent impossible to find in any other nanomaterial for in vivo MI (Figure 2).

These properties explain the use of IONPs in all major imaging techniques, with examples in OI [37], PAI [38], SPECT [39], PET [30] and MRI [36], and magnetic resonance imaging [35,40,41]. MRI deserves special mention since it is the most commonly used imaging technique with IONPs due to their magnetic properties. As it is well known, IONPs were initially developed for T_2_ contrast MRI. This approach benefits from the extremely large relaxivity values obtained with these nanoparticles, by virtue of their superparamagnetic behavior. Examples of this use are endless, from tumor diagnosis to cardiovascular and brain imaging [17,18,42]. However, despite being useful for some applications, the T_2_ (dark contrast) is a poor option for some applications where dark, endogenous contrast is usually found (due to the presence of other metals, bleeding, or in regions with very low density of protons). For this reason, research on IONPs for T_1_ (positive) contrast grew rapidly over the last years, with examples showing in vivo positive contrast using IONPs which previously could only be achieved with Gd-based probes [43,44]. It is known that a core size around 3 nm permits producing IONPs with a paramagnetic behavior rather than superparamagnetic, rendering IONPs with large effects on T_1_ relaxation time. On this aspect, the use of MW synthesis is also critical since it allows for the rapid synthesis of very small NPs [41,45]. 

### 1.3. Traditional Synthesis of Iron-Oxide Nanoparticles

Before studying the use of MW chemistry for the synthesis of IONPs, we briefly review traditional synthetic methods so that differences with MW-driven synthesis can be clearly seen. The synthesis method affects the size distribution, core shape, and surface properties, which further impact magnetic properties. For this reason, it must be carefully chosen depending on the final application. There are numerous routes to synthesize IONPs for biomedical applications (Table 1) [46]. Here, we show a very short description of most commonly used methods.

#### 1.3.1. Coprecipitation

Developed by Massart et al. in 1981, this method is based on the reaction of Fe^2+^ and Fe^3+^ in a 1:2 molar ratio, under inert atmosphere and in basic medium [47]. Under these conditions, magnetite NPs are synthesized, forming an ink-like precipitate. This procedure is represented by the following reaction:

Fe^2+^ + 2Fe^3+^ + 8OH^−^ → Fe_3_O_4_ + 4H_2_O. (1)

There are several parameters that can affect shape, size, magnetic properties, and colloidal stability of synthesized NPs. For instance, nanoparticles the same size can have very different magnetization values due to core impurities or surface effects [48,49]. Even though this method provides poor control over NP shape and size, this depends on reaction pH, ionic strength, temperature, and salt nature [50,51]. Organic acids [52,53,54], dendrimers [55], and polymers [56,57,58,59,60] are used to stabilize NPs obtained using this method.

#### 1.3.2. Thermal Decomposition

This method is based on the high-temperature decomposition of organometallic precursors in the presence of organic solvents and surfactants. The hot injection ensures instant nucleation and homogeneous NP growth; however, it generally renders NPs that are only dissolved in non-polar solvents. For this reason, an additional step is required to functionalize NPs and make them stable in physiological medium. It is a commonly used method, as it renders highly crystalline NPs with narrow size distributions. This is translated into large saturation magnetization values, making NPs obtained via this method ideal for T_2_ contrast in MRI.

**Figure 3:**
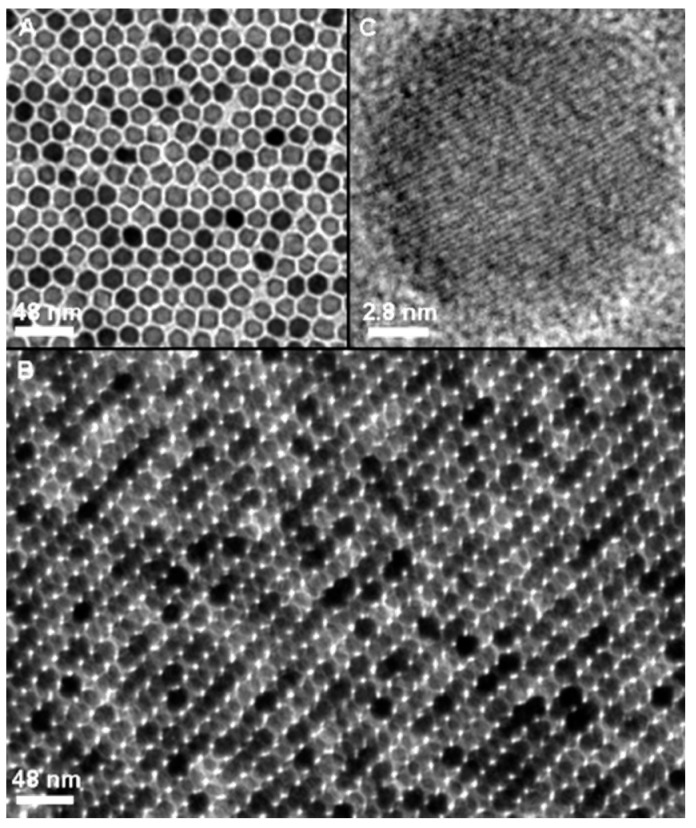
Transmission electron microscopy (TEM) images of IONPs synthesized by thermal decomposition. Reproduced with permission from Sun et al. [65].

There are examples of the use of different precursors, such as Fe(acac)_3_, Fe (oleate)_3_, FeO(OH), and FeCup_3_ in the presence of different surfactants and solvents [61,62,63,64]. The first IONP synthesis using thermal decomposition was carried out by Alivisatos et al., who reported the injection of FeCup_3_ (Cup: *N*-nitrosophenylhydroxylamine) in hot trioctylamine, yielding NPs from 4 nm to 10 nm, depending on synthesis temperature (from 250 °C to 300 °C) and iron precursor amount added. One of the most relevant variants of this synthesis route is the one described by Sun et al. in which Fe(acac)_3_ is decomposed using 1,2-hexadecanediol, oleylamine, and oleic acid as surfactants and diphenyl ether as solvent [65]. This reaction allows size control of NPs by varying reaction time and surfactant (Figure 3).

#### 1.3.3. Hydrothermal and Solvothermal Synthesis

Hydrothermal synthesis is based on aqueous media crystallization to form uniform, monodisperse NPs. To this end, temperature (between 130 °C or 250 °C) and/or pressure (0.3 MPa and 0.4 MPa) can be increased [66]. Under these conditions, Fe_3_O_4_ is oxidized in a controlled manner and mineralization of Fe^3+^ atoms takes place. FeCl_2_ [67,68], FeCl_3_ [69], and FeSO_4_ [70,71] are examples of iron precursors used in different syntheses carried out using this method. This synthesis route is chosen to synthesize IONPs with unconventional geometries. For instance, Wang et al. started with sodium oleate and iron chloride to synthesize superparamagnetic hematite nanocubes with an approximate size of 15 nm [69]. Titirici et al. prepared a general scalable hydrothermal synthesis for metal-oxide hollow spheres which they used to obtain hollow iron-oxide nanospheres ranging from 16 nm to 22 nm [71]. 

A variation of this synthesis route arose as a result of poor crystallinity of IONPs synthesized using the hydrothermal method. In the solvothermal method, aqueous media is replaced by organic solvents. Although reaction times are long, this method renders NPs with increased crystallinity and controllable size and shape [61,72,73,74,75]. 

#### 1.3.4. Pyrolysis

In spray pyrolysis, ferric salts and a reducing agent are dissolved in an organic solvent and then sprayed into a reactor in which the aerosol solute condenses into IONPs and the solvent evaporates [46,51]. NP sizes range from 2 nm to 7 nm, depending on the initial droplet size. This method yields small IONPs with homogeneous size distributions and appropriate magnetic properties for their potential use as contrast agents in MRI, which can be altered by changes in precursor concentration, pressure, and laser intensity [76]. Nevertheless, their application is strongly limited by the clustering these NPs undergo, coming from their increased surface energy [77]. For this reason, IONPs must be post-synthesis modified to improve colloidal stability and make them suitable for biomedical applications. Costo et al. performed an optimized acid post-treatment consisting of a reduction of NP surface disorder induced by a dissolution–recrystallisation process, rendering small aggregates with improved magnetic properties [78]. Malumbres et al. used triethylenegycol liquid medium to collect ultra-small magnetic nanocrystals prepared via hydrolysis in two different studies under different experimental conditions [77,79]. Zhang et al. polyethylene glycol (PEG)ylated IONPs prepared via pyrolysis using phase transfer to be used as nanocarriers for improved sonodynamic therapy [80]. Veintemillas-Verdaguer et al. produced inorganic nanocomposites composed of IONPs synthesized via laser-induced pyrolysis of aerosols encapsulated in carbon/silica or carbon matrices to be used as a contrast agent in MRI [81]. 

## 2. Microwave Synthesis of Nanoparticles

MW ovens are now an indispensable tool in modern organic synthesis. Nevertheless, it is known that this technique has the potential to contribute greatly to all areas of synthetic chemistry. In fact, the number of papers dedicated to MW-assisted synthesis of inorganic nanomaterials is growing rapidly. In this section, a few examples from different families of functional materials are selected in order to emphasize the versatility of the MW technique for accessing a large compositional diversity of inorganic compounds.

### 2.1. Metallic Nanostructures

Different MW-assisted routes are applied for the synthesis of different mono- and bimetallic NPs and nanostructures; some examples include gold (Au), silver (Ag), platinum (Pt), palladium (Pd), copper (Cu), or nickel (Ni), and combinations thereof. Generally, protocols for metal NP synthesis involve the chemical reduction of soluble metal salts in aqueous medium. Among a variety of metals, the MW-assisted preparation of Au nanostructures was most intensively investigated. This is due to their interesting properties and promising applications in various fields such as catalysis and biomedicine. Seol et al. [82] studied the formation kinetics of AuNP in MW-assisted synthesis. For that purpose, chloroauric acid (HAuCl_4_) was used as the gold source and trisodium citrate dehydrate (Na_3_Ct) was used as the reducing agent. During this study, it was shown that using high MW power to increase the temperature ramping rate (Rr) facilitates homogeneous nucleation, reducing NP size and improving uniformity. Using this method, they successfully produced uniform colloidal AuNPs with diameters of 12.0 nm in a few minutes. Bayazit et al. [83] developed a combined single-mode MW irradiation and microflow system capable of synthesizing AuNPs. Here again, HAuCl_4_ and Na_3_Ct were used as the gold source and reducing agent, respectively. However, using this new system, reaction variables (heat, temperature, reactant concentration, time, etc.) could be controlled during the course of the reaction, resulting in smaller particle sizes and size distributions while benefiting from a fast and high-throughput synthesis. Arshi et al. [84] reported a simple one-step MW irradiation method for the synthesis of AuNPs to use against *Escherichia coli* (*E. coli*). For this reaction, citric acid was used as the reducing agent and cetyl trimethyl ammonium bromide (CTAB) was used as the binding agent. Results yielded highly stable AuNPs with diameters of ~4.0 nm and ~1.0 nm depending on the MW irradiation time (40 s and 70 s, respectively_. Similarly, Thanh Ngo et al. [85] made use of MW heating for the synthesis of antibody functionalized AuNPs (13–15 nm in diameter) with antibacterial activity. HAuCl_4_ and Na_3_Ct were used as precursors for the synthesis of the AuNPs, which were posteriorly functionalized with an antibody against *E. coli* O157:H7, using *N*-hydroxy succinimide (NHS) and carbodimide hydrochloride (EDC) coupling chemistry. In addition to single-metal NPs, examples of MW-assisted synthesis of bimetallic NPs in aqueous solutions can be found in the literature. Cabello et al. [86] reported the microwave-assisted hydrothermal synthesis of bimetallic Pt–AuNPs, with different Pt/Au molar ratios, for the electro-catalytic oxidation of formic acid. These nanoalloys were synthesized via chemical reduction of the precursor salts hydrogen hexachloroplatinate (IV) hydrate (H_2_PtCl_6_·6H_2_O) and hydrogen tetrachloroaurate (III) trihydrate (HAuCl_4_·3H_2_O) using Na_3_Ct as the reducing agent.

Research on the MW-assisted synthesis of Ag nanostructures in aqueous solutions was also carried out. In most cases, water-soluble silver nitrate (AgNO_3_) was adopted as the silver source. For instance, Goel et al. [87] synthesized AgNPs to use against Gram-positive and Gram-negative bacterial strains. For this purpose, kappa-Carrageenan (κ-CRG), was used to formulate CRG-Ag nanocomposites through a facile MW synthesis approach. CRG-Ag NPs of size 50 ± 10 nm were obtained using CRG as a reducing and stabilizing agent.

**Figure 4:**
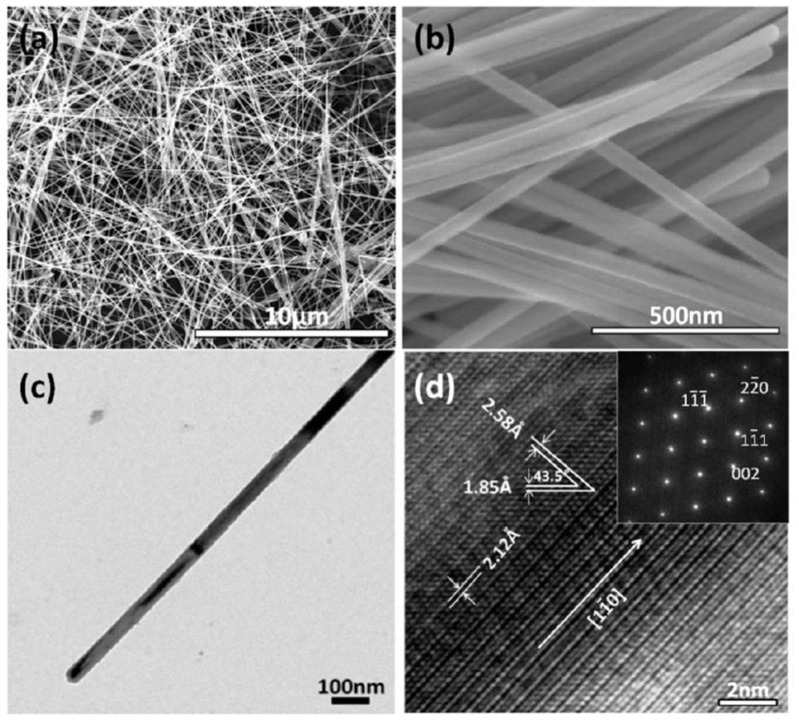
Structural and morphological characterization of Cu nanowires synthesized via a microwave (MW)-assisted method in aqueous solution. (**a**) Scanning electron microscopy (SEM) image; (**b**) magnified SEM image; (**c**) TEM image; and (**d**) high-resolution TEM image. Reproduced with permission from Reference [90].

In addition to the synthesis of Au and Ag nanostructures, the MW synthesis of other metallic NPs in aqueous solutions was investigated. Pal et al. [88] prepared PtNPs for the study of their catalytic behavior. For this synthesis, an aqueous solution of platinum chloride (H_2_PtCl_6_) was mixed with a solution of glucose and polyvinylpyrrolidone (PVP) acting as reducing and stabilizing agents, respectively. Furthermore, Mehta et al. [89] prepared PdNPs using palladium (II) chloride (PdCl_2_), glucose, and polyethylene glycol (PEG) dissolved in deionized water with microwave heating for only 20 s. Liu et al. [90] prepared single-crystalline Cu nanowires with MW heating using copper (II) chloride (CuCl_2_), ascorbic acid, and hexadecylamine in deionized water. The resultant Cu nanowires had an average diameter of 50 nm and lengths of longer than 10 μm (Figure 4).

MW energy is highly efficient and energy-saving in many chemical processes, and it is considered to be environmentally friendly for green synthesis of nanomaterials. In recent years, exciting progress was made in the development of environmentally friendly MW-assisted approaches for the preparation of metal nanostructures using biocompatible natural materials [91,92]. Eshghi et al. [93] synthesized AgNPs using AgNO_3_ as a silver source and *Juglans regia* leaf extract as both a reducing and stabilizing agent. This synthesis was carried out through a rapid one-step MW irradiation method, yielding AgNPs with mean sizes of 168 nm and a polydispersity index of 0.419, with high antibacterial activity toward *E. coli*. In another example, El-Naggar et al. [94] developed an eco-friendly microwave-assisted method for the rapid synthesis of core–shell Au–AgNPs. During this synthesis, curdlan (CRD) biopolymer performed the dual role of reducing and capping agent, yielding highly monodisperse spherical NPs with average sizes of 45 nm.

In addition to water, organic solvents can also be used for the preparation of metal NPs. Among these, polyols are especially popular due to their capability of acting as both a reducing agent and solvent. Saloga et al. [95] described a microwave-assisted polyol synthesis of ultra-small AgNPs. In their one-pot reaction, AgNO_3_ was used as the Ag source, while poly(acrylic acid) was used as the stabilizing agent, and polyethylene glycol was used as both the solvent and reducing agent. Furthermore, Wang et al. [96] and Duan et al. [97] also made use of the dual characteristics of polyethylene glycol for the synthesis of highly monodisperse PtNPs and Rh–AuNPs, respectively. Mishra et al. [98] synthesized chitosan-capped silver–dysprosium bimetallic NPs using an MW-assisted polyol reduction procedure. In this reaction, metal precursors, together with stabilizing chitosan aliquots, were mixed in a glycerol solution. NPs obtained through this method showed an average size of 10 nm with low polydispersity while displaying unique characteristics for applications in multimodal imaging.

Apart from polyols, examples of MW-assisted synthesis of metal NPs using other organic solvents can be found in the literature. Gutiérrez-Wing et al. [99] used toluene as both a solvent and reducing agent for the MW synthesis of AuNPs. Moreover, Li et al. [100] also exploited methanol’s dual characteristics for the synthesis of PtNPs and nanorods. Pujari et al. [101] used dimethyl sulfoxide (DMSO) as the solvent for the MW-assisted synthesis of hydrogen-terminated silicon NPs.

Finally, ionic liquids are also particularly suitable solvents for the preparation of metal nanostructures. These are known as excellent MW-absorbing agents due to their high ionic conductivity and polarizability. Bhawawet et al. [102] reported a very fast MW method to prepare oleylamine-capped AuNPs in a pyrrolidinium-based ionic liquid. Results yielded highly monodisperse AuNP with average sizes of 8–11 nm in diameter.

### 2.2. Metal Oxides

Considerable effort was devoted to the MW-assisted synthesis of nanostructures of various metal oxides due to their properties, high stability, and range of applications in many fields. Some examples include Fe_3_O_4_, ZnO, TiO_2_, CeO_2_, Al_2_O_3_, SnO_2_, BaWO_4_, or Co_3_O_4_. In most cases, a water-soluble metal salt is used as the metal source, a base is used to create an alkaline environment, and an additive or surfactant is often adopted to control the morphology and size of the product.

**Figure 5:**
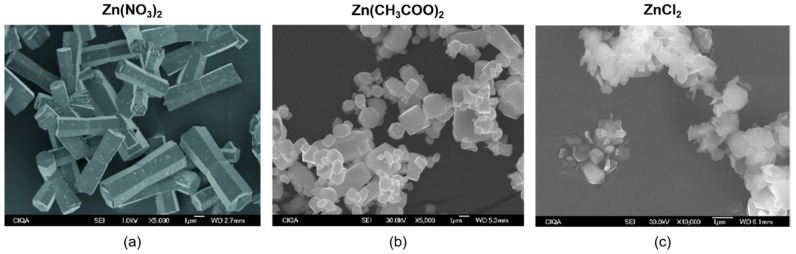
Field-emission (FE)-SEM images of ZnO nanostructures produced via MW-assisted synthesis at 140 °C and 600 W. The image shows the effect that using different precursor salts (**a**) Zn(NO_3_), (**b**) Zn(CH_3_COO)_2_, and (**c**) ZnCl_2_, has on the morphological development of ZnO during MW synthesis. Scale bar is 1 μm. Adapted with permission from Reference [103].

Barreto et al. [103] studied the use of three different precursor salts (Zn(NO_3_)_2_, Zn(CH_3_COO)_2_, and ZnCl_2_) and three different bases (NaOH, KOH, and NH_4_OH) for the microwave-assisted synthesis of ZnO NPs. During the reaction, the addition of an anionic surfactant (sodium di-2-ethylhexyl-sulfosuccinate—AOT) was also investigated. Results showed that the use of Zn(NO_3_)_2_ as the precursor salt yielded the highest purity of ZnO phase, with the addition of AOT resulting in smaller NPs (Figure 5). Hasanpoor et al. [104] successfully synthesized ZnO NPs with various morphologies (flower, needle, and spherical) and sizes using an MW-assisted hydrothermal procedure. Moreover, Yusof et al. [105] reported the synthesis of chitosan-capped ZnO NPs with antibacterial activity against *Staphylococcus aureus* and *E. coli*. A solution of Zn(NO_3_)_2_, NaOH, and chitosan was used for the preparation of the ZnO NPs, yielding uniformly distributed NPs with sizes ranging between 50 and 70 nm. In addition, Wojnarowicz et al. showed the high potential of MW synthesis to obtain ZnO structures with precisely controlled properties by synthesizing ZnO with controlled NP size [106] and ZnO NPs with controlled aggregate size [107] using an MW solvothermal synthesis.

TiO_2_ nanostructured materials also received strong attention, especially in photocatalysis. Regarding the MW-assisted synthesis of TiO_2_ nanostructures in aqueous solutions, Falk et al. [108] synthesized TiO_2_ NPs combining a conventional colloidal sol–gel reaction with an MW-assisted hydrothermal method. Firstly, the quick hydrolysis of titanium (IV) isopropoxide (TIPO) was produced in a mixture of deionized water and nitric acid. Subsequently, the solution was placed in a MW for hydrothermal reaction. Using this method, TiO_2_ NPs with average sizes of ~7 nm, a well-defined anatase crystalline phase, and high photocatalytic activity were successfully obtained.

High boiling points and high dielectric loss factors make organic solvents like ethylene glycol or benzyl alcohol perfect alternatives to an aqueous system. For this reason, several examples using MW-assisted techniques for the synthesis of metal oxides using organic solvents can be found in the literature. Dar et al. [109] developed a controlled synthesis of TiO_2_ nanostructures using an MW-assisted approach for their application in dye-sensitized solar cells. During their synthesis procedure, a thiobenzoate complex of titanium was dissolved in either ethanol or benzyl alcohol and subsequently exposed to MW irradiation. The resulting nanostructures appeared as nanospheres (NSs) when using ethanol and NPs when using benzyl alcohol. May-Masnou et al. [110] produced TiO_2_/Au NPs for gas-phase photocatalytic hydrogen generation. The fabrication of small anatase TiO_2_ NPs attached to larger anisotropic Au morphologies was carried out using a very fast and simple two-step MW-assisted synthesis. The TiO_2_/Au NPs were synthesized using PVP as a reducing, capping, and stabilizing agent through a polyol (benzyl alcohol) approach.

ZnO nanostructures were also synthesized using MW-assisted techniques and organic solvents. Kumar et al. [111] reported the synthesis of ZnO NPs via an MW-assisted hydrothermal technique used for the removal of methyl orange dye from wastewater. In this method, zinc (II) acetate was used as the zinc source, and dimethylformamide (DMF) was used as the solvent. Furthermore, Ambrožič et al. [112] studied the MW-assisted synthesis of ZnO NPs in non-aqueous media.

### 2.3. Metal Chalcogenides

The third kind of functional materials synthesized with MW-assisted methods are metal chalcogenides, more specifically, metal sulfides, metal selenides, and metal tellurides. To carry out the microwave-assisted synthesis of metal chalcogenide nanostructures, a metal salt is usually added into the solvent (aqueous or organic) as the metal source, while a source of sulfur, selenium, or tellurium is also added to provide ions, and an additive or surfactant is sometimes adopted to control the morphology and size of the product.

The MW-assisted rapid synthesis of metal sulfide nanostructures is interesting due to its advantages, especially in terms of processing time and control in size distribution [113]. Bharti et al. [114] used an MW-assisted hydrothermal method to synthesize CdS NPs for their use as photocatalysts and solar-cell devices. For that purpose, a cadmium sulfate (CdSO_4_8H_2_O) solution was added dropwise to a previously prepared solution of thiourea ((NH_2_)_2_CS), tertiary butyl alcohol (C_4_H_9_OH), cyclohexane (C_6_H_12_), and CTAB, yielding an average crystal size of bulk CdS NPs of 30–60 nm. Kim et al. [115] reported an MW-assisted synthesis of cadmium-free Cu–In–S/ZnS core/shell quantum dots (QDs) in aqueous phase. The resulting QDs showed an average size in the range of 3.5–3.7 nm and strong photoluminescence emission peaks. In addition, Shahid et al. [116] described an MW-assisted synthesis of ZnS QDs. In their reaction, ionic liquids were used as the MW-absorbing media and stabilizers. Two types of ionic liquids, imidazolium- and phosphonium-based, were used.

As compared to the microwave-assisted synthesis of nanostructured metal oxides and sulfides, the MW-assisted synthesis of metal-selenide nanostructures is less reported, possibly due to relatively high cost, less available selenide sources, and challenges in the preparation. The most studied metal-selenide nanostructure is the binary chalcogenide cadmium selenide (CdSe). Moghaddam et al. [117] described the MW-assisted synthesis of CdSe QDs. During their reaction, CdSe QDs were fabricated using selenium dioxide as a selenium precursor, 1-octadecene as a solvent and reducing agent, cadmium alkyl carboxylates or alkyl phosphonates as cadmium sources, 1,2-hexadecanediol as a cadmium complex stabilizer, and oleic acid as the resulting CdSe QD stabilizer. As a result, CdSe QDs with narrow size distributions and sizes ranging from 0.5–4 nm, depending on the cadmium source, were successfully obtained.

The preparation of nanostructured metal tellurides is even more challenging as compared to the synthesis of metal selenides. The reported work focused on the microwave-assisted synthesis of CdTe nanostructures, a very important semiconductor used as an infrared optical window and a solar-cell material. Ribeiro et al. [118] successfully synthesized water-soluble CdTe QDs capped with different stabilizing ligands by taking advantage of the MW dielectric heating, thus allowing rapid, uniform, and controlled nucleation and growth of nanocrystals. Using this method, highly luminescent, crystalline, and monodisperse nanocrystals, with suitable surface functionalities, were successfully obtained.

## 3. Microwave Synthesis of Iron-Oxide Nanoparticles

The MW-driven synthesis of IONPs is flexible enough to permit synthesizing almost any kind of iron-oxide-based nanostructure, with a variety of reaction conditions and, therefore, of sizes, shapes, and physicochemical properties, thereby explaining the success of this approach. This flexibility is achieved within an easy and completely reproducible experimental set-up which adds a key advantage over traditional methods. In this section, we summarize some of the key examples of the use of MW for iron-oxide NPs, focusing on reaction conditions and the most relevant physicochemical properties of the NPs. To classify the literature on this topic we focused on one of the most remarkable features of MW-driven synthesis—the reaction time. Microwave synthesis allows for the synthesis of IONPs in a wide range of timescales, from seconds to hours, something not easily achieved with traditional techniques.

### 3.1. From Seconds to Less Than Five Minutes

In this section, we explore the fastest examples published, i.e., those where NPs are produced in less than five minutes and, many times, in seconds. There are many advantages in using such a short time, but one where this is a key feature is when radiosiotopes are involved in the synthesis. In that case, when radioactivity is rapidly decaying, it is not possible to use time-consuming protocols, as they would result in the disappearance of the radioactivity. We see more examples of this in the applications section.

One of the first examples by Khollam et al. [119] made use of MW technology to synthesize submicron-sized magnetite agglomerates. They performed an MW hydrothermal synthesis using FeSO_4_, FeCl_3_, and NaOH. Reaction time and temperature were tuned in order to find the minimum for the formation of a single-phase material. They observed that 5 min and 90 °C were enough for the formation of Fe_3_O_4_ powder. X-ray diffraction (XRD) patterns of all synthesized powders indicated single-phase magnetite formation. Comparisons between different NaOH ratios and starting Fe^2+^ concentrations were tested. Large particles, from 150 nm to 200 nm, were obtained as result of smaller (~34 nm) particle agglomeration.

In a study carried out by Hu et al. [120] in 2011, magnetite, maghemite, and hematite NPs were synthesized using a hydrothermal MW-assisted method. Syntheses were performed in ethanol/water (*v*/*v* ½) with a reaction time between 2 and 6 min depending on the sample. Iron-chloride salts (FeCl_2_ and FeCl_3_) were dissolved in the medium, and sodium hydroxide was used as a base. The mixture was irradiated in an MW autoclave reactor whose power was adjusted to maintain constant pressure. Different parameter combinations were tried in eight different syntheses, varying the number of Fe^2+^ and Fe^3+^ and the NaOH amount. Samples were characterized using XRD, which showed well-defined patterns and extremely low backgrounds, meaning samples were crystalline. When Fe^3+^ was used as the sole precursor, hematite NPs were obtained. As Fe^2+^ amount increased, so too did NP crystallization. TEM analysis showed NPs ranging from 12 to 20 nm in core size, which agreed with values calculated from XRD data using the Scherrer equation, meaning NPs were spherical in shape. Presence of the different phases (hematite, maghemite, and magnetite) was confirmed using Fourier-transform infrared (FTIR) spectroscopy and X-ray photoelectron spectroscopy (XPS). Maghemite or magnetite was produced depending on drying process used.

Kooti et al. [121] synthesized maghemite NPs using Fe(acac)_3_ as an iron precursor, PEG-200 as a size controller, and NaCl as an MW absorber. This mixture was irradiated for 5 min at 1000 W. NPs were imaged by TEM and SEM, revealing an average core size of 13 nm and NP agglomeration.

Bano et al. [122] carried out a MW-assisted green synthesis of superparamagnetic NPs using fruit peel extracts as a biogenic reductant. FeCl_3_ was combined in water with fruit peel extracts of four different fruits: pomegranate, lemon, orange, and apple. Mixtures were subjected to MW irradiation at 800-W power in 30-s pulses. Obtained NPs were then surface-engineered via carbodiimide chemistry to functionalize them with PEG-6000 or succinic acid. Size ranged between 17 nm and 25 nm with an almost spherical morphology. NPs presented a good colloidal stability and water dispersibility and a large *r*_2_ value of 225 mM^−1^·s^−1^. Synthesized samples were shown to be hemocompatible at concentrations as high as 400 μg/mL. Their utility in photodynamic therapy was assessed in HeLa cells, which showed 23% decreased survival.

Sathya et al. [123] synthesized water-dispersible magnetite NPs by reducing Fe_2_(SO_4_)_3_ using sodium acetate as an alkali, PEG (5–6 kDa) as a capping ligand, and ethylene glycol as a solvent and reductant. Reactants were heated to 200 °C in 10 s in the MW and held at that temperature for different reaction times (from 10 s to 600 s). XRD data showed magnetite crystalline samples with NP core sizes between 9 nm and 24 nm. The hydrodynamic size of synthesized NPs, measured by dynamic light scattering (DLS), ranged from 35 nm to 141 nm, increasing with reaction time. Size distribution was narrow for all samples. Nanocluster size increased with reaction time from ~27 nm (10 s reaction time) to ~52 nm (600 s reaction time). Saturation magnetization values lay in the range between 32 emu/g and 58 emu/g.

Aivazoglou et al. [124] reported the synthesis of magnetite and maghemite NPs, studying the effect of reaction time, MW power, and capping agent on the properties of synthesized NPs. Different sets of syntheses were carried out. Firstly, no capping agent was used in syntheses of 2.5 min, while MW power was varied from 400 W to 800 W. In another set, PEG was used as a surfactant, and reaction time and MW power were varied from 400 W to 800 W and from 1 min to 5 min, respectively. The average size of NPs ranged from 10.3 nm to 19.2 nm, with a faceted and crystalline morphology, with no impurities. Magnetic measurements indicated samples were superparamagnetic. Their results showed that MW power and reaction time play a pivotal role in controlling NP size and maghemite presence, whereas ammonia concentration is not as relevant. PEG-assisted synthetic route rendered better results in terms of NP size and oxidation resistance.

### 3.2. Less Than 30 Minutes

In 2001, Liao et al. [125] reported the synthesis of amorphous Fe_2_O_3_ NPs via microwave irradiation by means of FeCl_3_ hydrolysis in aqueous solution in the presence of polyethylene glycol and urea. The reaction time was 10 min with fixed power at 650 W. NPs were studied by transmission electron microscopy (TEM), and cores sizes from 3 nm to 5 nm were observed. X-ray diffraction (XRD) revealed their amorphous structure.

In 2007, Hu et al. [126] synthesized α-Fe_2_O_3_ nanorings via a hydrothermal MW process. This one-pot synthesis was based on a previous method developed by Jiang et al. to grow Fe_2_O_3_ nanotubes without the use of microwave technology. FeCl_3_ and ammonium phosphate in aqueous solution were irradiated at 220 °C for 25 min. This process induced the generation of a hole of the primarily formed hematite nanodiscs, resulting in a nanoring formation. TEM revealed that more than 90% of the sample was made up of ring-like structures of an approximate outer diameter of 100 nm and an inner diameter of around 20 nm to 60 nm. These nanorings were intended to be useful for hydrogen peroxide biosensing in physiological solutions and gas sensing of alcohol vapor at room temperature.

Ai et al. [127] also synthesized rose-like nanocrystalline iron-oxide superstructures, composed of IONPs, for gas and magnetic sensing. They used ethylene glycol as a solvent, FeCl_3_ as an iron precursor, in addition to sodium acetate and a PEO–PPO–PEO block copolymer. This solution was irradiated at 160 °C for different times of 15, 30, and 60 min. Nanoparticles from 3 nm to 10 nm assembled to form the petals in the nanoroses. Saturation magnetization of the samples ranged between 34.5 emu/g and 37.1 emu/g, increasing with reaction time. All three samples showed a superparamagnetic behavior. These nanoroses presented high sensitivity and reversibility for gas sensing of ethanol vapor at room temperature.

**Figure 6:**
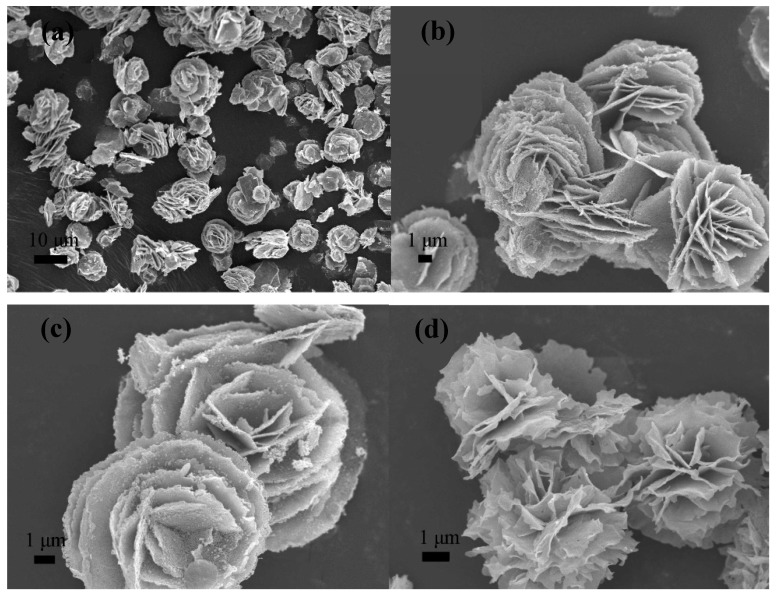
SEM images at low magnification (**a**), and high magnifications (**b**,**c**) of the Fe_3_O_4_ nanoroses obtained after 30 min of reaction; (**d**) the SEM image at low magnification. Reproduced with permission from Reference [127].

Magnetite and hematite NPs were synthesized by Wang et al. [128] using a microwave-assisted solution method. To synthesize magnetite NPs, FeCl_3_, PEG (20 kDa), and hydrazine were dissolved in water and introduced into the MW for 10 min and heated at 100 °C. To synthesize hematite NPs, the same procedure was followed; however, a mixture of hydrazine and hydrogen peroxide was used. SEM images showed magnetite NPs of less than 20 nm and wide size distribution, and hematite nanocrystals with ellipsoidal shape around 50 nm wide and 120 nm long (Figure 6). Saturation magnetization of the Fe_3_O_4_ NPs was 62.45 emu/g and ~10 emu/g for α-Fe_2_O_3_ NPs. Both samples presented small hysteresis loops.

Parsons et al. [129] conducted the synthesis of iron-oxide/oxyhydroxide nanophases via microwave irradiation. FeCl_3_ was titrated with NaOH and the mixture was introduced into the MW at seven different temperatures between 100 °C and 250 °C for 30 min. At lower temperatures (100 °C and 125 °C) iron oxyhydroxide chloride was synthesized, whereas, at temperatures above 150 °C, iron (III) oxide was synthesized. Average sizes of NPs, calculated from XRD data and the Scherrer equation, ranged from 17 nm to 33 nm. TEM images corroborated this and showed the NPs’ spherical morphology.

In 2012, Pascu et al. [130] carried out a study to compare microwave-assisted synthesized NPs (MW NPs) to those obtained from the thermal decomposition of organic precursors (TD NPs). Fe(acac)_3_ was used as an iron source and oleic acid was used as a stabilizer, with both dissolved in benzyl alcohol. The mixture was heated and stirred in the MW reactor at 60 °C for 5 min to completely resolve reactants. The mixture was posteriorly heated to 160 °C and held at that temperature for 15 min. Synthesized NPs were then solubilized in water using trimethylammonium hydroxide (TMAOH). NPs obtained were fully characterized and compared to NPs obtained via thermal decomposition. NP core size was calculated from XRD data using the Scherrer equation, yielding 4.6 nm for MW NPs and 5.4 nm for TD NPs. TEM revealed slightly larger, but very similar NP sizes for both samples (5.3 nm and 6.3 nm, respectively), with MW NPs more polydisperse to a small but negligible extent. Hydrodynamic size was measured by dynamic light scattering (DLS) and was 10.8 nm for MW NPs and 12.7 nm for TD NPs. Magnetic characterization by hysteresis loops revealed that MW NPs had a saturation magnetization of 60 emu/g and that of TD NPs was 62 emu/g. Both NPs presented good crystallinity. In 2015, this group performed a scale-up IONP microwave-assisted thermal decomposition synthesis [131]. A single-mode MW unit, yielding 4.5 mL and 22 mg of Fe_2_O_3_, was scaled up in a multi-mode MW unit of up to 500 mL, corresponding to 2.61 g of Fe_2_O_3_. High NP yields were obtained (80%) and magnetic and physicochemical properties of the NPs were not compromised.

MW-synthesized water-dispersible superparamagnetic IONPs were used by Carenza et al. [132] to safely label endothelial progenitor cells. Fe(acac)_3_ was dissolved in benzyl alcohol and irradiated at 60 °C for 5 min. Posteriorly, temperature was raised to 180 °C and held for 10 more minutes. This yielded uncoated NPs which were posteriorly coated with citric acid in an additional step as an electrostatic stabilizer. TEM imaging revealed that NPs presented a roundish lobular shape with an average core size of 7.2 nm and 18% polydispersity, meaning size distribution was narrow. Electron diffraction showed defined diffraction rings corresponding to a maghemite spinel structure. Hysteresis loops showed that NPs had a superparamagnetic behavior and a saturation magnetization of 60 emu/g at 300 K. Hydrodynamic NP size was measured by DLS, yielding 14 nm with a polydispersity index of 0.2. The sample was incubated with different biological media, and it was found to aggregate in endothelial growth medium (EGM-2), but not in fetal bovine serum (FBS). Cellular uptake of NPs in both states (dispersed and aggregated NPs) was investigated by TEM, in which differences in the size and number of cytoplasmic vessels could be seen. Uptake was sevenfold more efficient for systems with large NP aggregates, without compromising cell viability, morphology, and functionality.

In 2013, Kozakova et al. [133] synthesized IONPs for high-frequency applications using a solvothermal MW-assisted method. Ethylene glycol was selected as the reaction and reduction medium. FeCl_3_ was used as the iron salt, and two ammonium salts were used (ammonium carbonate and ammonium bicarbonate); water addition was selected as an extra variable for one of the syntheses. Reactions were carried out in an MW pressurized reactor, while the temperature was set to 220 °C, and the reaction time was 30 min. XRD revealed the presence of maghemite and/or magnetite in sample, which were not distinguishable using this technique. TEM images showed spherical morphologies for all samples. The sample nucleated with ammonium carbonate contained a significant amount of crystalline impurities and a size of ~130 nm, with a broad size distribution. Samples prepared using ammonium bicarbonate were smaller in size, with an average size of 40 nm for the one with no water content and 10 nm for the one containing water, while both were highly monodisperse. Hysteresis loops revealed that samples prepared with ammonium carbonate presented higher saturation magnetization values (75 emu/g) and a small hysteresis loop was observable (65 Oe coercivity) for samples with no water content, whereas saturation magnetization was 52 emu/g with a decreased coercivity (2 Oe) for the water-containing sample. Saturation magnetization decreased to 42 emu/g for the sample prepared with ammonium bicarbonate, which also presented a negligible coercivity of 2 Oe. Posteriorly, in 2015, this same group carried out another study to elucidate the effect of MW heating on processes and reactions occurring during NP synthesis, developing a simple method that allows the tailoring the properties of products obtained [134]. Nano and submicron particles were prepared via a solvothermal MW-assisted method. Ethylene glycol and FeCl_3_ were also selected as a solvent and iron precursor, respectively, in this study. However, they added an extra nucleating agent to the ones used in the previous work—ammonium acetate. They also played with sample water content (2 mL and 4 mL). The reaction time was 30 min and three different synthesis temperatures were tried (200 °C, 210 °C, and 220 °C). Particles ranging from 20 nm to 100 nm based on single crystals or crystalline assemblies were obtained upon varying the nucleating agent. A variation of the reaction medium could be modified by water addition, which resulted in a threefold reduction in NP size and, therefore, in magnetic behavior changes to superparamagnetic and ferromagnetic properties. In the same year, they also carried out a two-step MW-assisted thermal decomposition technique to synthesize magnetic needle-like IONPs [135]. The metallic precursor was obtained via a solvothermal method using iron (II) sulfate heptahydrate and oxalic acid in a mixed water/ethanol solvent. This mixture was introduced into a synthesis MW reactor for 30 min at 100 °C. The obtained yellow precipitate was then filtered and sealed in a tube that was introduced into a domestic MW oven at 750 W for 15 min. This enabled reaching extremely high temperatures in very short times. Temperature measured immediately after decomposition using a contactless pyrometer was ~450 °C. The obtained product was characterized using XRD, which showed that the sample was crystalline. Scanning electron microscopy (SEM) was used to study morphology and size. The prepared particles had a long needle-like shape, a diameter of less than 1 μm, and a length of 20 μm, yielding a high aspect ratio. Magnetic property characterization with a vibrating sample magnetometer (VSM) revealed a ferromagnetic behavior with a saturation magnetization of 43 emu/g and a coercivity of 124 Oe.

Kalyani et al. [136] prepared IONPs using MW technology at two different temperatures (45 °C and 85 °C) to study how NPs can be tuned with synthesis temperature. A mixture of FeSO_4_ and ammonium hydroxide in aqueous solution was irradiated for 30 min at the two set temperatures. XRD revealed magnetite crystalline cores of 10 nm for NPs synthesized at 45 °C and 13.8 nm for those synthesized at 85 °C. Magnetization curves of both samples showed very low coercivity and remanence values. Saturation magnetization values were 67 emu/g and 72 emu/g, respectively.

MW-assisted thermal decomposition was selected by Liang et al. [137] to synthesize monodisperse magnetite NPs. Fe(acac)_3_ was mixed with oleic acid, oleylamine, and octadecene. The mixture was heated to 200 °C in 10 min in the MW. This temperature was held for another 10 min. Synthesis yield was 90.1%, which was a high value compared to traditional thermal decomposition (~20% yield). Subsequently, temperature was risen to 250 °C in 5 min and held for five more minutes. NP characterization revealed that the mean core size was ~6 nm and that NPs were highly monodisperse. Core crystallinity was verified using XRD. VSM measurements revealed that the NPs presented a superparamagnetic behavior and a saturation magnetization of 76 emu/g. A large transverse relaxivity value (*r*_2_) was measured (172 mM^−1^·s^−1^).

Guru et al. carried out two different studies in study in 2016 to investigate the effect of different anions [138] and the effect of different glycols [139] in the formation of IONPs in a 10-min MW synthesis. In the first study, they prepared magnetite, maghemite, hematite, and iron-oxide hydroxide NPs using different precursor salts (FeSO_4_, FeCl_3_, and Fe(NO_3_)_3_). They obtained NPs ranging from 19.4 nm to 80 nm in size. In the second work, they studied the effect of using ethylene glycol, polyethylene glycol, or propylene glycol in NP synthesis. The obtained NPs ranged from 11.7 to 46.7 nm in core size. Thermal studies revealed that NP stability increased with the molecular weight of glycols used.

Sangaiya et al. [140] studied how tin-doping affects IONP properties. IONP and Sn-doped IONP were synthesized with varying amounts of Sn (from 10 wt.% to 50 wt.%). Hexadecyl trimethyl ammonium bromide (HTAB) was used as a surfactant. Samples were irradiated for 15 min in a MW reactor at a radiation frequency of 2.41 GHz. Changes in Sn levels led to morphology changes; low doping rendered rhombus-shaped platelets of around 40 nm, and, as Sn-doping increased, NP morphology became spherical and the core size decreased to approximately 20 nm.

**Figure 7:**
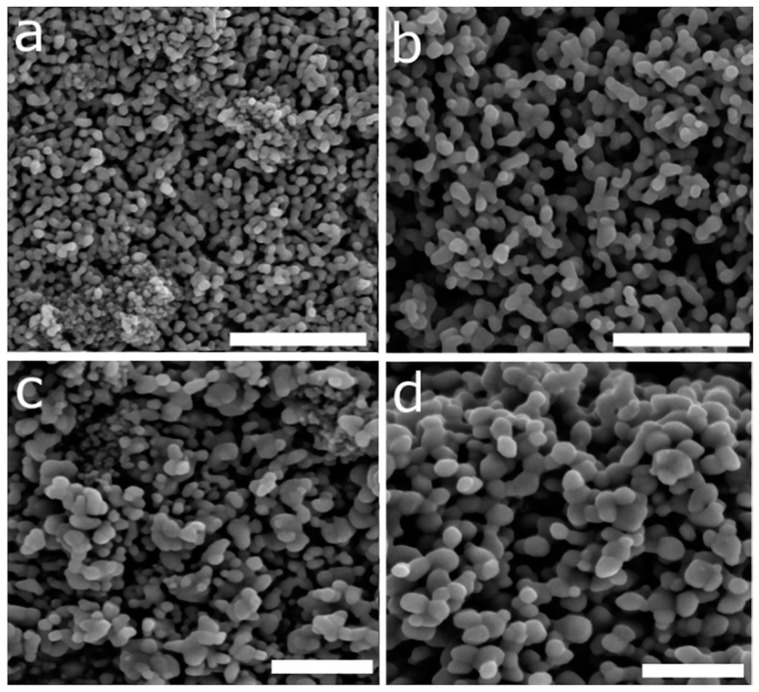
FE-SEM images of the (**a**,**b**) nanoshards, and (**c**,**d**) rhombohedral NP films synthesized by Hammond et al. after calcination, measured using a thin film coating (10 nm) of chromium. Scale bars depict 1 mm. Reproduced with permission from Reference [141].

In 2017, Hammond et al. [141] prepared IONPs for photochemical solar water splitting using a MW-driven solvothermal method with deep eutectic solvents (DES), a new kind of mixed solvent system formed upon the complexation of hydrogen-bonding salts and neutrally charged species. Fe(NO)_3_ was added to the selected DES, and the mixture was irradiated for 10 min at three different temperatures (100 °C, 150 °C, or 200 °C). The prepared iron-oxide nanostructures varied in phase, size, and morphology as synthetic conditions were varied (Figure 7). Using pure DES, small nanoparticulates were obtained, whereas, when hydrated DES was used, nanoshards (at 150 °C) or large rhombohedral NPs (at 200 °C) were synthesized. Syntheses performed at 150 °C yielded maghemite nanostructures; however, those that took place at 200 °C rendered hematite NPs. Small spherical NPs of around 3 nm aggregated into a sponge-like composite of ~50 nm and presented a superparamagnetic behavior. Large rhombohedral structures synthesized with hydrated DES presented a ferromagnetic behavior.

### 3.3. One Hour and More

Co-ferrite nanoparticles were synthesized by Bensebaa et al. [142] in 2004 via a microwave-assisted coprecipitation protocol. A mixture of 1,2-propanediol, sodium acetate, cobalt (II) acetate, and iron (III) chloride was heated to 160 °C at 30 °C/min and held at that temperature under vigorous stirring for 60 min. TEM showed that the synthesized ferrite material was composed of regular NPs of around 5 nm in core size. The XRD pattern obtained showed similar features to those corresponding to cobalt ferrite preparations. Thermogravimetric analysis (TGA) suggested that a small amount of acetate molecules remained adsorbed on the surface of the NPs. The value for saturation magnetization obtained at 10 K was 105 emu/g, which is slightly higher than that for bulk CoFe_2_O_4_ (90 emu/g).

A microwave-assisted hydrothermal method was chosen by Li et al. [143] to synthesize IONPs. FeCl_3_, ammonium acetate, and trisodium citrate were dissolved in ethylene glycol and posteriorly introduced into the MW reactor. Precursors were heated to 260 °C in 15 min and held at that temperature for 2 h. XRD data showed crystalline NPs of an estimated size of 13.56 nm according to the Scherrer equation. No impurity peaks were observed, indicating the high purity of the Fe_3_O_4_ NPs. Morphology and structure were studied by TEM, which revealed Fe_3_O_4_ aggregates ranging from 40 nm to 70 nm composed of smaller NPs (9 to 13 nm in core size). Saturation magnetization was measured performing hysteresis loops. Saturation magnetization was 41 emu/g and the sample showed a superparamagnetic behavior, with no hysteresis at 300 K. However, at 10 K, NPs showed a ferromagnetic behavior, with a coercivity value of 252 Oe.

Jiang et al. [144] prepared iron-oxide nanocubes via thermal decomposition of iron oleate in the presence of oleic acid via microwave-assisted solvothermal synthesis. Fe(oleate)_3_ was driven by a MW digestion system. The solution was transferred to a Teflon-lined digestion vessel and irradiated for 6 min at 400 W. Samples obtained after this step were low crystalline spheres of about 6 nm in core size with superparamagnetic behavior. After aging at 180 °C for 10 and 20 h, crystalline α-Fe_2_O_3_ nanocubes of sizes ranging between 13 nm and 28 nm formed through Oswald ripening, in which small NPs dissolved and redeposited onto the surface of bigger NPs. Saturation magnetization increased from 8 emu/g to 30 emu/g after aging at 180 °C for 20 h, and nanocubes became ferromagnetic.

Multi-core IONPs for magnetic hyperthermia were synthesized by Blanco-Andújar et al. [145] Citric-acid-coated NPs were obtained via the microwave-assisted coprecipitation of FeCl_2_ and FeCl_3_, which were previously heated to 60 °C in the presence of citric acid and sodium carbonate. The solution was irradiated at 60 °C for 10 or 60 min, at two different powers (50 W or 300 W). NP morphology and size were studied with TEM. Core sizes obtained range from 13 nm to 17 nm, with a spherical shape. The hydrodynamic size of samples ranged from 49.5 nm to 141 nm, with low polydispersity indexes. The magnetic properties of all samples were fairly similar, with all samples presenting superparamagnetic behavior at 300 K and a saturation magnetization of ~72 emu/g. They concluded that the best heating performance was obtained with large cores (17 nm) forming relatively small ensembles (65 nm).

Using MW-assisted synthesis in organic solution, Lastovina et al. [146] synthesized maghemite NPs via thermal decomposition of Fe(acac)_3_ in the presence of oleylamine and 1,2-hexadecanediol and varying amounts of oleic acid. Precursors were irradiated at 120 °C for 60 min in the first step, and at 185 °C for 90 min in the second step. NPs obtained ranged from 3.5 nm to 5.3 nm in size, calculated from XRD data and the Scherrer equation. TEM showed that the presence of oleic acid reduced NP aggregation compared to pure oleylamine. Magnetic behavior showed a clear trend from superparamagnetic to paramagnetic behavior as the amount of oleic acid in synthesis increased. Only samples with 20% oleic acid presented hysteresis, as the NPs were larger.

In 2018, the same group developed an MW-assisted method to synthesize Sm^3+^-doped maghemite NPs as potential contrast agents for MRI [147]. Lastovina and co-workers carried out a one-pot polyol coprecipitation synthesis using FeCl_2_, FeCl_3_, SmCl_3_, and NaOH in an ethylene glycol/polyethylene glycol solution (10/1 mL) in the presence of gold NPs. The mixture was introduced into the MW for 3 h at 200 °C. X-ray fluorescence analysis of the sample with gold nanoparticles showed 2.95 wt.% and 0.95 wt.% concentrations of Sm and Au, respectively (with respect to Fe), along with scarcely distributed gold NPs and thin covering layer of partially oxidized glycol molecules over iron oxide. TEM imaging showed round NP morphology and an average size of 4.5 nm, matching data obtained by XRD. The size coincided with gold NPs added, proving a size equivalence of composite components. NPs presented a superparamagnetic behavior with a saturation magnetization of 33 emu/g. Cell viability studies were carried out to test NP toxicity. HeLa cells were used in live/dead assays, revealing that NPs had a minimal cytotoxic effect.

## 4. Applications of Microwave-Produced Iron-Oxide Nanoparticles in Molecular Imaging

### 4.1. Iron-Oxide Nanoparticles as T_2_ Contrast Agents

Several examples that use MW synthesized IONPs as T_2_ MRI contrast agents are described in the literature. As we previously mentioned, the use of this type of contrast can be problematic depending on the pathology and tissue under study. Researchers should be careful when using this type of contrast in cases where endogenous hypointense areas are expected. An example of this can be lung imaging, where the low density of water protons and the air–tissue interface naturally produces low-intensity areas; another example occurs if there exists bleeding or metal deposits such as Ca^2+^.

In spite of these problems, it is a perfectly valid approach for many applications. For example, Brollo et al. [148] optimized an MW-assisted method for the synthesis of IONPs from a solid precursor for use as T_2_ MRI contrast agents. In order to carry out the MW-assisted synthesis, a unique precursor, solid iron oleate, was previously synthesized via a slightly modified technique from a patented protocol [149]. The effect of different key MW parameters such as solvent, heating ramp, and iron concentration had in the synthesis of IONPs was evaluated for optimization purposes. After several trials, the group concluded that dibenzyl ether was the optimal solvent. In addition, they concluded that the optimal heating ramp was 3.75 °C/min, the ideal iron concentration was 4 mg Fe/mL, and the oleic acid/Fe ratio was 5, giving rise to the most uniform NPs. They produced two MW samples of IONPs. However, one sample used dibenzyl ether as a solvent (MwE8) and the other used benzyl alcohol (MwA8). Once prepared, solutions were introduced into the MW and stirred at 600 rpm while the temperature increased 3.75 °C/min until 250 °C, maintained for 1 h. Further functionalization to make the NPs stable in aqueous media was necessary. For that purpose, they exchanged oleic acid with dimercaptosuccinic acid (DMSA). All peaks present in their XRD patterns corresponded to crystallographic magnetite or maghemite planes. TEM images showed core sizes of 6.9 nm for MwE8 and 7.3 nm for MwA8, only slightly different from those calculated with XRD, indicating the single-core character of the NPs. Zeta potential was −34.4 mV for MwE8 and −27.6 mV for MwA8, which was given by the negatively charged character of DMSA. Hydrodynamic sizes were 23 nm and 68 nm for MwE8 and MwA8, respectively, which increased as the amount of coating increased on the NP surface, as observed in the thermogravimetric analysis (TGA). Magnetic measurements showed that IONPs presented nearly superparamagnetic behavior at room temperature (RT), showing rather low coercive fields. Saturation magnetization showed a relatively small value of 55 emu/g Fe for MwE8 and 100 emu/g Fe for MwA8. Measured relaxivity values yielded an *r*_2_/*r*_1_ ratio of 8 for MwE8 and 28.1 for MwA8, making them good candidates for T_2_ MRI.

**Figure 8:**
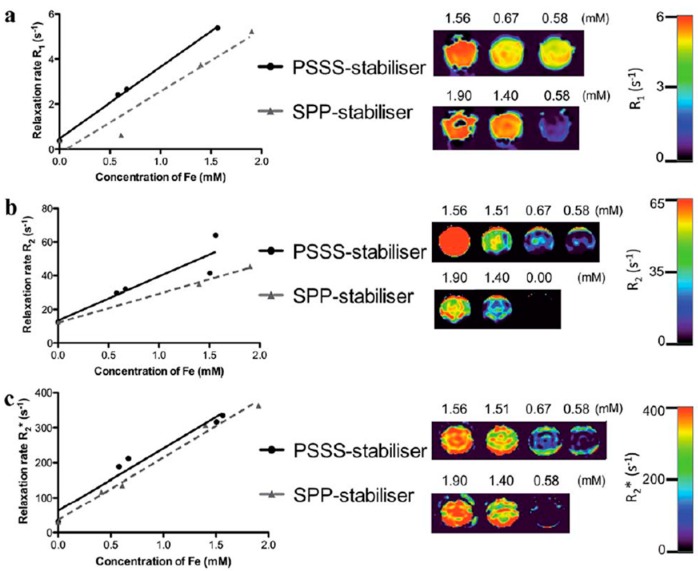
Relaxivities of the Fe_3_O_4_-poly(sodium 4-styrenesulfonate) (PSSS) (straight line) and Fe_3_O_4_-sodium polyphosphate (SPP) (dashed line) nanocomposites measured at 3 T and 20 °C. Scatter plots show correlations between measured (**a**) R_1_, (**b**) R_2_, and (**c**) R*_2_ values of the nanocomposites and iron concentrations measured using inductively coupled plasma mass spectrometry (ICP-MS). The relaxation rates (R_1_, R_2_, and R*_2_) were determined at 3 T using T_1_, T_2_, and T*_2_ mapping sequences, respectively, and aqueous solutions between 0 and 2 mM of the contrast agents. Pearson’s correlation coefficient values ranged from 0.92 to 0.99. Phantom MRI images of the formulations show R_1_, R_2_, and R*_2_ maps in color scale. R_1_, R_2_ and R*_2_ values increase with increasing concentrations of contrast agents (highest concentration on the left). Reproduced with permission from Reference [150].

Williams et al. [150] described a single-step MW-assisted method for the synthesis of multifunctional nanocomposites comprising superparamagnetic iron-oxide cores, a polyelectrolyte stabilizer, and an organic dye, for imaging applications. Polyelectrolytes were poly(sodium 4-styrenesulfonate) (PSSS) and sodium polyphosphate (SPP). For the preparation of the magnetic composites, FeCl_3_ and FeCl_2_ were firstly dissolved in deoxygenated water. Subsequently, the polyelectrolyte stabilizer (either PSSS or SPP) was dissolved in the iron solution and heated to 80 °C. Following this, an ammonia solution was injected into the solution, which was stirred for 20 min before transferring to the MW cavity to be heated at 150 °C for 20 min. For fluorescently labeled samples, rhodamine B was dissolved with the polyelectrolyte stabilizer (PSSS) before mixing it with the iron solution. TEM images showed aggregation of the polyelectrolyte functionalized NPs; however, at higher magnifications, it was observed that each agglomerated region consisted of numerous discrete NPs clustered together, with core sizes of 13.4 nm and 10.1 nm for Fe_3_O_4_-PSSS and Fe_3_O_4_-SSP, respectively. Nevertheless, the shape of Fe_3_O_4_-PSSS NPs appeared better defined than the Fe_3_O_4_-SSP NPs. DLS measurements of both samples gave a hydrodynamic mean size of ~100 nm. Zeta potential was in the range of −40 mV for both samples, with both showing excellent water stability for over six months. Acquired relaxivity values showed an *r*_2_/*r*_1_ ratio of 8.18 for Fe_3_O_4_-PSSS and 6.21 for Fe_3_O_4_-SSP, making them good candidates for T_2_ MRI. This was confirmed by phantom MR images that showed reduced signal intensity as Fe concentration was increased (Figure 8). The effect of the stabilizer on cell toxicity was examined using cell viability studies on suspensions of the polyelectrolyte-stabilized NPs co-incubated at increasing concentrations with a range of cell lines. Regardless of the stabilizer employed, all NP suspensions tested were found to be non-toxic to a variety of mammalian cell lines. Moreover, to demonstrate uptake into mammalian cells, fluorescently labeled samples were prepared via the addition of rhodamine B to the PSSS polyelectrolyte solution before particle precipitation. NP uptake was observed in the cytoplasm of UKF-NB-3 neuroblastoma cells.

**Figure 9:**
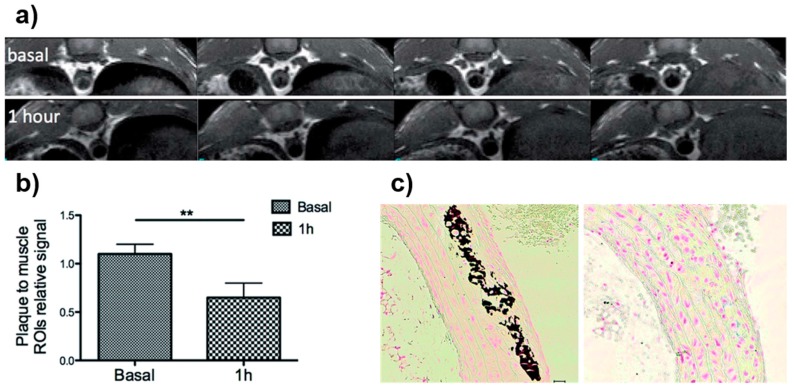
(**a**) In vivo MRI of ApoE^−/−^ mouse prior to (top) and one hour after the intravenous (i.v.) injection of neridronate-functionalized IONPs (bottom); (**b**) plaque to muscle relative signal intensity prior to (basal) and one hour after the i.v. injection of neridronate-functionaliszed IONPs; (**c**) histology of plaque sections stained with von Kossa and Prussian blue for mineralization and iron detection, respectively, scale bar is 22.2 mm. Adapted with permission from Reference [18].

Our group described a fast and reproducible MW-driven process to synthesize neridronate-functionalized IONPs for the in vivo visualization of atherosclerotic plaque by T_2_-weighted MRI [18]. Neridronate possesses a high binding affinity toward Ca^2+^, which allowed its use as a targeting moiety toward the calcium vesicles in the atheroma plaque for atherosclerosis diagnosis. Firstly, oleic-acid-coated IONPs (OA-IONPs) were synthesized in the MW. To that end, a mixture containing Fe(acac)_3_, oleic acid, oleylamine, and 1,2-dodecanediol in benzyl alcohol was stirred at 60 °C for 2 min at 300 W. After that, temperature was increased from 60 °C to 180 °C and the mixture stirred for 20 min. The core size of the OA-IONPs, as given by TEM, was 3.7 nm. Furthermore, the hydrodynamic size obtained with DLS was 7.5 nm. In order to make these NPs stable in water, a direct chemical modification of the surfactant, from oleic acid to azelaic acid, was carried out using two MW steps of 9 min each. This procedure rendered azelaic-acid IONPs with a hydrodynamic size of 30 nm. Moreover, TEM showed no NP aggregation upon coating modification and a core size of 4.9 nm. The FTIR spectra of these NPs showed the expected changes due to the breaking of the oleic-acid chain. In addition, relaxivity values of azelaic-acid IONPs were measured to determine their performance as T_2_-weighted MRI contrast agents; these were 15.3 mM^−1^∙s^−1^ for *r*_1_ and 90.7 mM^−1^∙s^−1^ for *r*_2_. Finally, the neridronate-functionalized IONPs were synthesized; for that purpose, a final modification consisting on the amide formation between the amine group in neridronate and the carboxylic groups in the azelaic-acid molecules was carried out using EDC/sulfo-NHS chemistry. TEM images showed a homogeneous distribution with core sizes of 5.5 nm. Furthermore, hydrodynamic size was 40 nm with a narrow size distribution. In addition, the incorporation of the new functional group was confirmed both by FTIR and energy-dispersive X-ray (EDX) analysis. Relaxivity values calculated for these samples were 11.2 mM^−1^∙s^−1^ for *r*_1_ and 93.3 mM^−1^∙s^−1^ for *r*_2_, demonstrating their suitability as T_2_-weighted MRI contrast agents. The affinity of the synthesized NPs toward Ca^2+^ was analyzed by the incubation of the NPs with different calcium solutions. Results showed that the value of the T_2_ relaxation time increased linearly with the amount of Ca^2+^ due to the formation of NP clusters, confirming that the neridronate binding capacity was not lost during functionalization. In vitro studies confirmed the cellular uptake of the NPs by flow cytometry and bright-field microscopy. Furthermore, cytotoxicity experiments clearly showed no remarkable cytotoxic effects even at high NP concentration. In vivo MRI experiments, using ApoE^−/−^ mice, showed the appearance of an hypointense signal in the MR images of the aorta of the atherosclerotic prone mice after NP injection (Figure 9). Furthermore, the difference in intensity before and after NP injection was quantified showing a clear reduction one hour after NP injection. Finally, ex vivo experiments were performed to confirm the obtained results. Ex vivo MRI together with histology confirmed the accumulation of IONPs in the aortic plaque and their colocalization with the Ca^2+^.

The combination of complementary imaging techniques allows exploiting the best of both worlds. PET/MRI, for instance, combines the excellent sensitivity of PET with the outstanding spatial resolution MRI presents. For this reason, Wong et al. [33] developed a rapid MW synthetic procedure of ^64^Cu core-doped IONPs for dual-mode PET/MR imaging. FeCl_2_, FeCl_3_, and CuCl_2_ were used. Dextran was selected as a surfactant, and NH_4_OH was used as a reducing agent. The mixture was introduced into the MW for different reaction times and powers, ranging from 5 to 15 min and 150 W to 30 W, respectively. They performed syntheses with and without Cu-doping. NPs obtained ranged from 45.7 nm to 65.3 nm in hydrodynamic size and from 3.1 nm to 3.3 nm in core size. They determined that NPs produced at 300 W were the optimal ones and selected them for further characterization. An incorporation efficiency of 10% was achieved. Relaxometry revealed that there was a significant increase in transversal and longitudinal relaxivities triggered by Cu incorporation into the NP cores. Magnetic characterization revealed superparamagnetic behavior and a saturation magnetization of 16 emu/g for non-doped IONP and 12 emu/g for Cu-doped NPs. FTIR further confirmed the presence of dextran in the surface of the magnetite copper-doped NPs. Once they found the optimal reaction time and power, synthesis with radioactive ^64^CuCl_2_ was performed. The reaction time was 1 min, and the power was 300 W. The hydrodynamic size of NPs obtained was 35.3 nm and the core size measured by TEM was 4.2 nm. The transversal relaxivity value was 100 mM^−1^·s^−1^. ^64^Cu presence was verified by gamma counting after purification to remove free radiometals. They obtained a 33% radiolabeling yield.

### 4.2. Iron-Oxide Nanoparticles as T_1_ Contrast Agents

Gold-standard commercial contrast agent types for T_1_ imaging are still gadolinium-based, which are proven to be toxic for patients with renal conditions. For this reason, the need for a biocompatible contrast agent for T_1_ imaging and easy surface functionalization motivated the quest for IONPs suitable as T_1_ contrast agents.

In 2015, our group presented an MW-assisted synthesis of fluorescein-labeled iron-oxide NPs for T_1_ MRI and cell labeling [45]. Fluorescein isothiocyanate (FITC)–dextran was used as a surfactant, rendering a fluorescent signal and providing a further anchor for posterior functionalization. FeCl_3_ was chosen as the iron precursor and hydrazine hydrate was chosen as the reducing agent. The mixture was irradiated at 100 °C for 10 min, taking 54 s to reach the desired temperature. This approach yielded NPs with a hydrodynamic size of 21.5 nm with a polydispersity index (PDI) of 0.18, measured by DLS. Surface charge, indicated by the zeta potential, was −15.8 mV. Core size was observable by TEM and was ~2.5 nm. The difference between hydrodynamic and core size was explained by the large polymeric coating on the surface of the NPs, as observable in TGA, with an 80% mass loss at 300 °C. FTIR spectra confirmed the presence of dextran in the surface of NPs and core maghemite composition. Magnetic characterization by superconducting quantum interference device (SQUID) revealed a saturation magnetization of 17.2 emu/g and no hysteresis. Relaxivity values, measured at 1.5 T, were 5.97 mM^−1^·s^−1^ (longitudinal) and 27.95 mM^−1^·s^−1^ (transversal), yielding an *r*_2_/*r*_1_ ratio of 4.7. Cell labeling studies were carried out using mouse adult fibroblasts (MAFs). They were incubated with different NP concentrations and studied under fluorescence microscopy and T_1_ MRI. Cells with the highest NP concentration showed the highest intensity of bright contrast. This proved NPs to be positive MR contrast agents. Epifluorescent images were acquired, showing that fluorescence intensity increased with iron concentration. In vivo magnetic resonance angiography was carried out in mice to test the contrast agent potential of NPs. NPs were injected intravenously, and images were acquired 6, 30, 60, and 90 min after. NPs allowed the visualization of main vascular architecture, visible even 90 min post injection. By virtue of their small size and biocompatible surface coating, they remained in circulation much longer than other NPs, proving to be excellent candidates for blood-pool applications and as a platform for targeted molecular imaging.

In 2017, our group presented a one-step MW-assisted synthesis of IONPs for T_1_ MRI, in which relaxometric properties can be tuned by adjusting coating composition and thickness while preserving core size, crystallinity, and magnetic moment [35]. By modifying the coating, these IONPs can also be used as T_2_ contrast agents, enabling the full-range application of MRI. Synthesis was carried out using FeCl_3_ as a precursor, citric acid as a surfactant, and hydrazine hydrate as a reducing agent. Samples were irradiated for 10 min at nine different temperatures, from 60 °C to 140 °C, in 10 °C intervals. The hydrodynamic size of samples was measured using DLS, revealing that it ranged from 4.9 nm to 21 nm, increasing with synthesis temperature. All samples had narrow size distributions. Four samples were selected for further characterization: those synthesized at 80 °C, 100 °C, 120 °C, and 140 °C. Electron microscopy showed very similar core sizes for all samples, ranging from 3.5 nm to 4.5 nm. XRD data showed a slight progression of crystallinity with synthesis temperature, with high similarity for samples synthesized at 120 °C and 140 °C. Zeta potential measurements lay around −30 mV, as expected for citrate-coated NPs. FTIR spectroscopy revealed that cores were composed of maghemite. To further inquire into differences between core size and hydrodynamic sizes, TGA was carried out. Samples synthesized at 80 °C, 100 °C, and 120 °C presented similar profiles, typical for citrate-coated NPs; however, the sample synthesized at 140 °C showed two clear mass loss steps, typical in a bilayer formation. Further analysis showed this sample contained a much larger amount of organic molecules than the other three samples. Mass spectrometry analysis revealed that the sample at 140 °C contained aconitic acid in the surface (in addition to citric acid), which is a product of citric acid dehydration. Magnetic characterization revealed superparamagnetic behavior of all samples with saturation magnetization increasing as synthesis temperature increased (from ~20 emu/g to ~70 emu/g). Relaxometric values were measured for the nine initial samples at 1.5 T. Again, a progression with synthesis temperature could be observed. As temperature increased, so did *r*_1_ value, while maintaining low *r*_2_ values, indicating that the samples were ideal for T_1_ contrast. This changed at 140 °C, where *r*_2_ value increased pointedly, yielding a high *r*_2_/*r*_1_ ratio, meaning this sample was more suitable as a T_2_ contrast agent. Once biocompatibility of samples was assessed in vitro in mouse adult fibroblasts (MAFs), in vivo imaging MR studies were carried out in murine models to check our contrast change hypothesis. The sample synthesized at 120 °C was selected for T_1_ MR angiography and the sample at 140 °C was selected for T_2_ liver MRI. As expected, angiography was eased by the sample at 120 °C, allowing an accurate visualization of mouse vasculature and confirming its potential as a blood-pool contrast agent. The sample synthesized at 140 °C presented typical signal characteristics and biodistribution of a T_2_ NP-based contrast agent. Furthermore, citric-acid coating paves the way for further NP functionalization for specific targeting.

**Figure 10:**
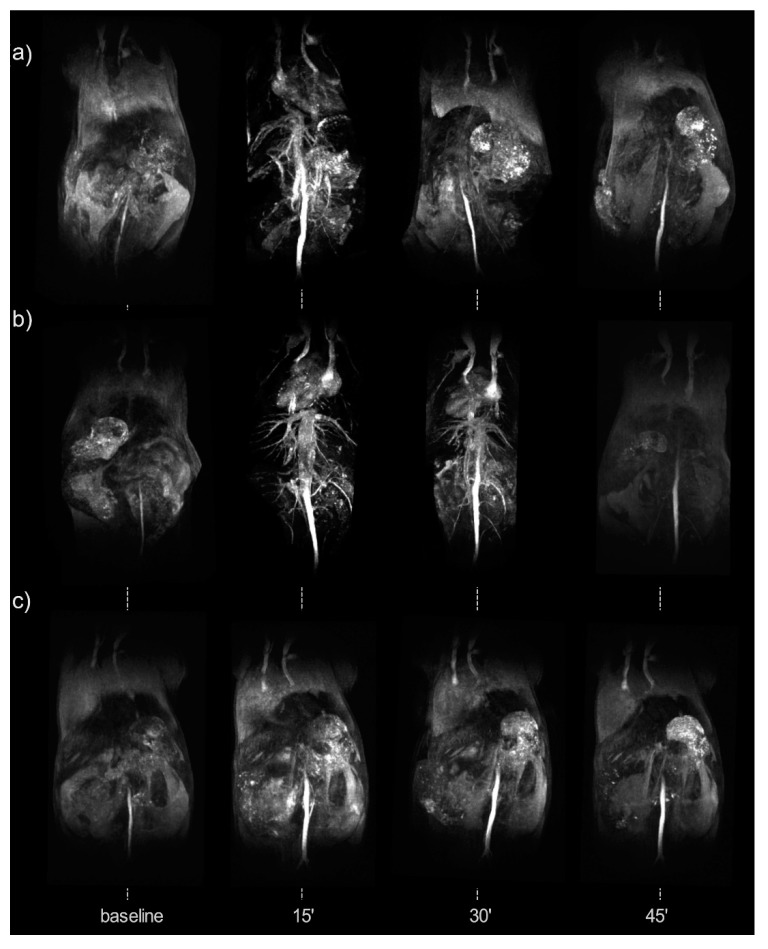
MRI (*T*_1_-weighted imaging) body angiography in healthy mice, before and after the intravenous injection of (**a**) 1.7% Cu-doped NPs, (**b**) 4% Cu-doped NPs, and (**c**) 28% Cu-doped NPs. Reproduced with permission from Reference [41].

We recently studied how doping these aforementioned NPs synthesized at 120 °C with different copper amounts affected their physicochemical, magnetic, and relaxometric properties [41]. The synthetic procedure was the same as mentioned before. However, to the previous reactants (FeCl_3_, citrate trisodium, and hydrazine hydrate), we added a varying amount of CuCl_2_. Samples were irradiated at 120 °C for 10 min. We performed three different syntheses in which we obtained IONPs with 1.7% mol, 4% mol, and 28% mol Cu doping. Hydrodynamic size analysis revealed narrow size distributions for all samples. The sample with 1.7% mol Cu doping was the smallest, while those with 4% mol and 28% mol Cu doping had a very similar size distribution to IONPs synthesized at 120 °C. No relevant zeta potential changes were observable, with values around −34 mV for all samples. FTIR spectra showed expected bands, confirming maghemite core composition. High-angle annular dark field scanning transmission electron microscopy (STEM-HAADF) imaging showed well-formed and disperse NPs. The sample with 1.7% mol Cu doping presented the smallest core size (3.2 nm), whereas 28% mol doping and IONPs showed the largest values (4.4 nm and 4.2 nm, respectively). Energy-dispersive X-ray (EDX) analysis reinforced the inductively coupled plasma mass spectrometry (ICP-MS) analysis, whereby we encountered an increase in Cu/Fe ratio as % mol Cu doping increased. Magnetic characterization revealed superparamagnetic behavior of all samples. Saturation magnetization values decreased as Cu doping amount increased with respect to IONPs (from 76 emu/g for IONPs to 43.8 emu/g for 28% mol Cu-doped sample). Relaxometric characterization revealed samples with 1.7% and 4% mol Cu doping had improved longitudinal relaxivity (13.6 mM^−1^·s^−1^ and 15.7 mM^−1^·s^−1^, respectively) with respect to IONPs (11.9 mM^−1^·s^−1^). Transverse relaxivity for these two samples was also larger; however, the *r*_2_/*r*_1_ ratio was still pretty low. The sample with 28% mol Cu doping yielded lower transverse and longitudinal relaxivity values. In vivo MR angiography experiments were conducted with the three Cu-doped samples in order to assess their T_1_ signal potential. Differences between NP samples were clearly observed. Angiography with 4% mol Cu-doped sample provided high-quality images with fine vascular details up to 30 min post NP injection. The sample with 1.7% mol Cu doping yielded positive contrast 15 min post NP injection, but weakened after 30 min, which was attributable to differences in *r*_1_ and *r*_2_/*r*_1_ values. In view of the excellent performance of the 4% mol Cu-doped sample, actively targeted molecular imaging was carried out using this sample and IONP alone to test differences in contrast behavior. A classical targeting experiment was designed, functionalizing both samples with RGD peptide via amide formation with a citric-acid coating of NPs. This peptide targets vascular angiogenesis toward integrin α_v_β_3_. After characterization, and once functionalization of NPs with the peptide was confirmed, samples were injected in mouse breast cancer allograft models. T_1_ MRI was performed 1 h post intravenous injection of both samples (Figure 10). Differences were remarkable; passive accumulation in the tumor could be discarded, as non-functionalized NPs of both 4% mol Cu-doped IONPs and IONPs were used as controls, and no significant accumulation could be observed. The image acquired after IONP-RGD injection showed a modest increase in tumor signal. This changed for the 4% mol Cu-doped sample, as a clear increase in signal could be observed and quantified (signal increased by 30%) by virtue of its larger *r*_1_ value with respect to IONPs.

Combining IONPs with PET radionuclides seems a feasible approach to generate new hybrid systems for multimodal imaging. In our case, our choice was ^68^Ga radiometal. It has a suitable half-time (68 min) for in vivo imaging and avoids the need for a cyclotron nearby, as it is produced in a radionuclide generator. We successfully incorporated ^68^Ga into the nucleus of the MW-synthesized NPs using a similar MW procedure to the ones mentioned above [151]. To the FeCl_3_ and selected surfactant (citric acid or dextran), we added ^68^GaCl_3_ eluted from the generator in situ. Hydrazine hydrate was added, and the sample was introduced into the MW and heated at 100 °C or 120 °C for 10 min.

In 2016, we presented a fast and reproducible synthesis of ^68^Ga core-doped IONPs for PET/MR imaging of angiogenesis in tumors [30]. In this case, dextran was selected as a surfactant, and synthesis was carried out at 100 °C. Obtained NPs were fully characterized prior to their use in vivo. Radiolabeling yield was 93.4%. DLS unveiled that NPs were 20.6 nm in hydrodynamic size. TEM imaging showed extremely small cores (average of 2.2 nm) and no aggregation. Surface composition was assessed by FTIR and TGA, showing maghemite core composition and consistently high dextran surface coating, which ensures NPs colloidal stability. Superparamagnetic behavior of the NPs was confirmed by SQUID, revealing a saturation magnetization of 19.2 emu/g. Longitudinal and transverse relaxivity values were 5.7 mM^−1^·s^−1^ and 22.2 mM^−1^·s^−1^, respectively. Incorporation of ^68^Ga into the core of the NPs was confirmed by both indirect radioactive routes and directly by EDS and X-ray fluorescence (XRF). Once characterization was complete, NPs were functionalized with RGD peptide via EDC/sulfo-NHS chemistry and amide formation. These functionalized NPs were then injected in vivo in tumor-bearing mice. One hour post intravenous injection, PET/CT images were acquired, revealing remarkable tumor accumulation and typical liver and spleen accumulation of the NP probe, which was posteriorly confirmed by a biodistribution study using a gamma counter. Control experiments were carried out with non-functionalized NPs, and blocking experiments discarded passive tumor accumulation.

In a posterior work in 2017, ^68^Ga-doped NPs were synthesized using citric acid as a surfactant instead of dextran to visualize inflammation processes in vivo [152]. Synthesis was carried out at 100 °C. Radiolabeling yield was 92%, yielding a specific activity of 7.1 GBq/mmol Fe. Core size was 2.7 nm, measured by TEM. Hydrodynamic size was measured by DLS, found to be 14.5 nm. The longitudinal relaxivity value was 6.8 mM^−1^·s^−1^ and the transversal relaxivity value was 15.9 mM^−1^·s^−1^. Testing of PET and MRI signals by performing phantom studies with ^68^Ga-doped NPs proved the successful incorporation of ^68^Ga into the core of the NPs, with PET signal increasing proportionally with NP concentration. These NPs were functionalized covalently with a neutrophil-specific peptide (cFLFLF) via amide-bond formation by EDC/sulfo-NHS chemistry. The hydrodynamic size of functionalized NPs increased to 83.3 nm, due to hydrophobicity of the peptide. However, no aggregation was observed, meaning colloidal stability was not compromised. Peptide attachment was confirmed by FTIR and by a decrease in zeta potential negativity (from −31.5 mV to −14.6 mV). Cytotoxicity experiments in primary hematopoietic cells and mature neutrophils revealed no significant toxicity nor alteration in neutrophil function. The efficiency of these NPs as a neutrophil-specific radiotracer was assessed by in vivo PET/CT imaging experiments. Functionalized NPs were injected in vivo in acute lung inflammation and chronic inflammation murine models. PET imaging revealed the high targeting efficiency of the functionalized NPs, allowing the acquisition of high-quality images of neutrophil recruitment. Specificity for neutrophils was ensured using a neutrophil-depleted model.

**Figure 11:**
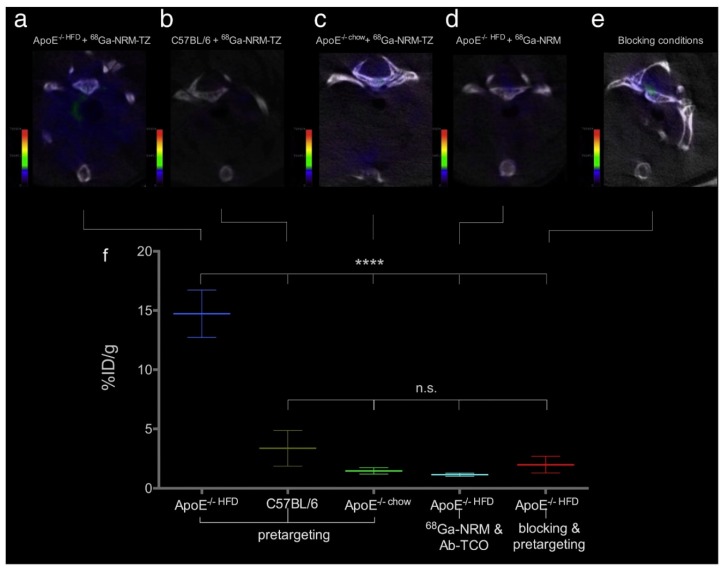
(**a**–**e**) Axial positron-emission tomography (PET)/computed tomography (CT) images taken 24 h post injection of E06-TCO and 1 h post-injection of NPs: (**a**) ApoE^−/−HFD^ mice injected with ^68^Ga-NRM-TZ; (**b**) C57BL/6 mice injected with ^68^Ga-NRM-TZ; (**c**) ApoE^−/−chow^ mice injected with ^68^Ga-NRM-TZ; (**d**) ApoE^−/−HFD^ mice injected with ^68^Ga-NRM; (**e**) ApoE^−/−HFD^ mice injected with antibody (Ab), prior to Ab-TCO and ^68^Ga-NRM-TZ. (**f**) Radioactivity uptake in mice aortas for the five conditions, detected with a gamma counter 2 h post injection and expressed as the percentage of injected dose per gram of tissue (mean ± standard deviation; statistical analysis by two-tailed *t*-test, *N* = 3–5; ****, *p* = 0.0009). Reproduced with permission from Reference [153].

Moreover, we recently used the citrate-coated ^68^Ga core-doped nano-radiomaterials (^68^Ga–NRM) synthesized at 120 °C for atherosclerosis plaque detection using a pretargeted approach by PET and MRI [153]. Pretargeted imaging is the use of biorthogonal probes that selectively accumulate upon reaction with a previously modified biomolecule in vivo. We chose tetrazine and *trans*-cyclooctene, a biorthogonal pair involved in tetrazine ligation. We selected E06 as a targeting vector. E06 is an antibody targeting oxidized low-density lipoprotein (LDL), present in atherosclerotic plaques. This antibody was modified with the *trans*-cyclooctene. ^68^Ga core-doped NPs were synthesized as previously mentioned, at 120 °C for 10 min under MW irradiation. Radiolabeling yield was 92%. Hydrodynamic size was 14.7 nm, measured by DLS. STEM-HAADF imaging revealed disperse and well-formed NP cores of 2.9 nm. FTIR analysis confirmed citrate presence on the surface of the NPs and maghemite core composition. NPs were then functionalized with tetrazine, the second component of the bioorthogonal reaction (^68^Ga-NRM-TZ). Functionalization did not compromise NP stability, barely increasing the hydrodynamic size to 15.5 nm. It did not modify relaxometric properties either. The longitudinal relaxivity value was 7.1 mM^−1^·s^−1^, maintaining a low *r*_2_/*r*_1_ ratio. In vivo imaging experiments were carried out in atherosclerotic mouse models (ApoE^−/−^ mice). The approach was as follows: *trans*-cyclooctene-modified (TCO) E06 was firstly injected; 24 h later, when antibody was cleared from bloodstream, freshly prepared tetrazine-functionalized ^68^Ga doped-NPs were injected. Then, 1 h post NP injection, PET images wee acquired, revealing significant NP accumulation in atherosclerotic lesions (Figure 11). Non-functionalized NPs and C57BL/6 (healthy) mice were used in control experiments to ensure probe specificity. Given the poor MRI sensitivity, monitoring and locating IONPs using T_1_ is non-trivial, especially at high magnetic fields. Thus, aortas where excised and imaged by MR. Images clearly revealed NP presence in the atherosclerotic plaque of ApoE^−/−^ mice, demonstrating the feasibility of the dual PET/MR approach.

## 5. Conclusions

The use of MW chemistry for the synthesis of NPs offers numerous advantages. As we tried to show in this comprehensive review, flexibility of reactions conditions, along with the reproducibility and fixed experimental set-up, is ideal to reproducibly produce NPs for biomedical applications. This is particularly true for IONPs. This nanomaterial is being used in all major imaging techniques and, for some of them, with relevant clinical applications. For these reasons, we believe the use of MW synthesis is particularly important since it is not user-dependent; therefore, consistent batch production is ensured.

## Figures and Tables

**Table 1:** Iron-oxide nanoparticle (IONP) synthesis methods, and their advantages and disadvantages.

Synthesis Method	Advantages	Disadvantages	Time Range
Coprecipitation	Large amount of NPs synthesized in short amount of time Hydrophilic NPs High efficiency (96–99.9% yields) Simple method	Amorphous NP cores; poor crystallinity Poor control over NP size and shape Broad size distribution of NPs Long reaction times Basic pH required	Hours–days
Thermal Decomposition	Crystalline NP cores NP size control Monodisperse NPs High yields obtained (~80%)	Hydrophobic NPs obtained; additional step required to solubilize them in physiological media Long reaction times Requires organic solvents	Hours–days
Hydrothermal and Solvothermal Synthesis	Controllable NP size Monodisperse NPs Hydrophilic NPs Scalable	Long reaction times to obtain monodisperse NPs Special reactors or autoclaves usually needed (high temperature and high pressure)	Hours–days
Pyrolysis	Controllable NP size Monodisperse NPs High rate production	NPs obtained by this method tend to aggregate; post-synthesis modifications required to improve NP colloidal stability Impurities	Hours
Microwave Synthesis	Tunable NP size Monodisperse NPs Hydrophilic NPs High efficiency Short reaction times	Microwave reactor required	Seconds–hours

## Abbreviations

α-Fe_2_O_3_	Hematite
γ-Fe_2_O_3_	Maghemite
[^18^F]FDG	^18^F-fluorodeoxyglucose
AuNPs	Gold nanoparticles
CRD	Curdlan
CT	Computed tomography
DES	Deep eutectic solvent
DLS	Dynamic light scattering
DMSA	Dimercaptosuccinic acid
DMSO	Dimethyl sulfoxide
EDX	Energy-dispersive X-ray spectroscopy
FBS	Fetal bovine serum
Fe_3_O_4_	Magnetite
FTIR	Fourier-transform infrared spectroscopy
IONPs	Iron-oxide nanoparticles
MI	Molecular imaging
MPI	Magnetic particle imaging
MRI	Magnetic resonance imaging
MW	Microwave
NPs	Nanoparticles
NRM	Nano-radiomaterial
NS	Nanospheres
OI	Optical imaging
PAI	Photoacoustic imaging
PET	Positron emission tomography
PDI	Polydispersity index
QDs	Quantum dots
r_1_	Longitudinal relaxivity value
r_2_	Transversal relaxivity value
RT	Room temperature
SWNT	Singe-walled carbon nanotube
SEM	Scanning electron microscopy
SPECT	Single-photon computed tomography
SQUID	Superconducting quantum interference device
STEM-HAADF	High-angle annular dark field scanning transmission electron microscopy
T_1_	Longitudinal relaxation time
T_2_	Transversal relaxation time
TCO	*trans*-cyclooctene
TGA	Thermogravimetric analysis
TEM	Transmission electron microscopy
VSM	Vibrating sample magnetometer
XRF	X-ray fluorescence spectroscopy
XPS	X-ray photoelectron spectroscopy
XRD	X-ray diffraction
